# Body Composition in Resectable Non‐Small Cell Lung Cancer Patients With Preoperative Sarcopenia: A Propensity‐Matched Analysis

**DOI:** 10.1002/jcsm.70230

**Published:** 2026-02-12

**Authors:** Zhu‐zhu Wang, Jin‐yu He, Rui‐feng Tang, Tian Zhang, Le Luo, Yu‐han Zhang, Jing Zhang, Xiao‐qing Lv, Jing‐fang Hong

**Affiliations:** ^1^ The First Affiliated Hospital of Anhui Medical University Hefei Anhui China; ^2^ School of Nursing Anhui Medical University Hefei Anhui China; ^3^ Department of Thoracic Surgery, the First Affiliated Hospital of USTC, Division of Life Sciences and Medicine University of Science and Technology of China Hefei Anhui China; ^4^ Nursing International Collaboration Research Center of Anhui Province Hefei Anhui China

**Keywords:** bioelectrical impedance analysis, body composition, non‐small cell lung cancer, propensity score matching, sarcopenia

## Abstract

**Background:**

Sarcopenia is a prognostic factor in patients with early‐stage non‐small cell lung cancer (NSCLC). Previous studies have focused on muscle loss, yet comprehensive body composition (BC) alterations in this population remain poorly characterized. This study aimed to delineate BC alterations using bioelectrical impedance analysis (BIA) in early‐stage NSCLC patients with preoperative sarcopenia, with a specific focus on sex‐specific disparities and the identification of independent BC factors associated with sarcopenia.

**Methods:**

In this case‐control study, 460 patients with Stage I–II NSCLC were initially enrolled from two tertiary hospitals in Anhui, China. Sarcopenia was diagnosed preoperatively based on Asian Working Group for Sarcopenia 2019 criteria. Multifrequency BIA was performed within 48 h before surgery. Propensity score matching (PSM) at a 1:4 ratio was applied to balance covariates (age, sex, height, physical activity, nutritional status, clinical stage, histology, extent of resection and diabetes). Multivariable logistic regressions were used to examine the associations between BC parameters and sarcopenia.

**Results:**

After PSM, 47 sarcopenic and 162 nonsarcopenic patients were well‐matched. Sarcopenic patients exhibited systemic depletion beyond reduced muscle mass, including lower body fat mass (BFM: 12.70 vs. 18.60 kg), body cell mass (BCM: 23.90 vs. 29.10 kg), bone mineral content (BMC: 2.22 vs. 2.52 kg = 0.002), basal metabolic rate (BMR: 1151.00 vs. 1340.50 kcal) and elevated extracellular water/intracellular water ratio (ECW/ICW: 0.64 vs. 0.63; all *p* < 0.001). Distinct sex‐specific phenotypes were identified: Sarcopenic females demonstrated coordinated reductions in muscle, fat and minerals (appendicular skeletal muscle mass [ASM]: 13.10 vs. 15.43 kg; BFM: 11.20 vs. 19.20 kg; percent body fat [PBF]: 25.41% vs. 32.88%; BMC: 2.05 vs. 2.26 kg; all *p* < 0.001), whereas males exhibited isolated muscle loss with preserved adiposity and minerals (PBF: 23.04% vs. 24.29%, *p* = 0.463; BMC: 2.64 vs. 2.80 kg, *p* = 0.141). In the fully adjusted model, ASM (OR = 0.03, 95%CI: 0.01–0.08), BFM (OR = 0.81, 95%CI: 0.75–0.87) and BMR (OR = 0.95, 95%CI: 0.94–0.97) were independent factors associated with sarcopenia. Sex significantly modified associations for waist–hip ratio, soft lean mass, FFM and PBF (all *p* for interaction < 0.05). Sensitivity analyses based on sarcopenia severity supported the robustness of the primary findings.

**Conclusions:**

Preoperative sarcopenia in early‐stage NSCLC involves multicompartment depletion accompanied by cellular dysfunction and metabolic impairment, exhibiting distinct sex‐specific phenotypes. BIA provides a practical tool for multidimensional BC assessment, necessitating to integrate it into clinical workflows and developing sex‐tailored prehabilitation strategies.

## 1 Introduction

Lung cancer remains the leading global oncologic challenge, with GLOBOCAN 2022 reporting 2.48 million new cases and 1.82 million annual deaths worldwide, where non‐small cell lung cancer (NSCLC) accounts for 85% of diagnoses [1]. Beyond its direct and devastating impact on survival, NSCLC and its associated treatments significantly disrupt body composition (BC), notably accelerating skeletal muscle loss and dysfunction—a condition termed sarcopenia [2, 3]. Contrary to conventional perception, substantial BC alterations, including fat depletion and sarcopenia, are not limited to advanced disease stages [4]. Emerging evidence indicates that such adverse changes occur even in patients with early‐stage, potentially curable NSCLC, likely driven by cancer‐related systemic inflammation, tumour‐induced metabolic reprogramming (e.g., heightened catabolic activity) and treatment‐related adverse effects such as anorexia, nausea and fatigue [3, 5, 6]. The early emergence of sarcopenia may considerably impair physiological resilience, compromise postoperative recovery and diminish long‐term survival [3, 7, 8, 9, 10]. For instance, studies have demonstrated that NSCLC patients with pre‐existing sarcopenia prior to lung resection exhibit a lower 5‐year overall survival rate (79.9%) compared to those who develop new‐onset sarcopenia after surgery [3]. Thus, accurate preoperative BC assessment, particularly for sarcopenia, is paramount for risk stratification in early‐stage resectable NSCLC.

Although computed tomography (CT)‐derived skeletal muscle index (SMI) at the third lumbar vertebra (L3) is considered the gold standard for muscle mass quantification, its clinical utility is constrained by radiation exposure, cost and limited accessibility [11, 12]. Routine preoperative thoracic CT protocols for NSCLC patients typically do not encompass the L3 region [2]. In contrast, bioelectrical impedance analysis (BIA)—characterized by its non‐invasiveness, rapid operation, absence of radiation and minimal training requirements—offers a practical alternative endorsed by both the Asian Working Group for Sarcopenia (AWGS 2019) and the European Working Group on Sarcopenia in Older People (EWGSOP2) for clinical application [12, 13]. Validation studies have shown that BIA exhibits high agreement with dual‐energy X‐ray absorptiometry for estimating fat free mass (FFM) and percentage body fat (PBF), with correlation coefficients ranging from 0.90 to 0.98, supporting its utility particularly in screening contexts [14]. Beyond mass estimation, BIA provides a comprehensive physiological profile through parameters such as phase angle (PhA), which serves as a marker of cellular integrity and health [15], the extracellular water to intracellular water ratio (ECW/ICW), which reflects fluid balance and hydration status [16], and basal metabolic rate (BMR), an estimate of resting energy expenditure [16]. These measures offer clinically actionable insights into metabolic dysregulation and subcellular dysfunction, often occurring before overt muscle depletion becomes apparent [17].

Despite this potential, few studies have fully leveraged the multidimensional capabilities of BIA for comprehensive BC profiling in preoperative patients with early‐stage NSCLC and sarcopenia, while adequately controlling for key confounders and accounting for sex‐specific biological differences [2, 18, 19]. This gap is particularly relevant given growing evidence highlighting sexually dimorphic pathways in cancer‐associated muscle wasting [20]. To address these limitations, this study aimed to (i) comprehensively characterize BC alterations using BIA in early‐stage NSCLC patients with preoperative sarcopenia; (ii) examine sex‐specific differences in these alterations; and (iii) identify independent BC factors associated with sarcopenia. For this purpose, sex‐stratified, multidimensional BIA‐derived BC profiling was comprehensively analysed in rigorously matched patients with early‐stage (I–II) NSCLC. The findings from this comprehensive BC profiling aim to identify novel modifiable targets, thereby informing the development of personalized prehabilitation strategies designed to enhance physiological resilience and improve clinical outcomes.

## 2 Material and Methods

### 2.1 Study Design and Population

This case‐control study was conducted using the baseline data from a prospectively collected dataset of consecutive adults (≥ 18 years) with Stage I–II NSCLC who underwent video‐assisted thoracoscopic surgery (VATS) at two tertiary hospitals in Anhui Province, China: The First Affiliated Hospital of Anhui Medical University (recruited from October 2024 to July 2025) and The First Affiliated Hospital of University of Science and Technology of China (recruited from January to July 2025). The exclusion criteria were as follows: extreme anthropometrics (height < 145 cm or body mass index ≥ 50 kg/m^2^), contraindications to BIA (implanted electronic devices, limb amputations or severe edema), acute systemic illnesses, life expectancy < 6 months due to non‐oncologic comorbidities, inability to complete functional assessments or incomplete clinical data.

The study protocol was approved by the ethics committees of both institutions (No. 82240150; No. 2025KY‐200) in compliance with the Declaration of Helsinki and the Chinese Ethical Guidelines for Medical and Health Research Involving Human Subjects. Written informed consent was obtained from all participants prior to data collection. The methodology adhered rigorously to the Strengthening the Reporting of Observational Studies in Epidemiology (STROBE) guidelines [21].

### 2.2 Data Collection

Data for patient‐reported outcomes, anthropometry, functional tests and BC analysis were collected prospectively within 48 h prior to surgery. Demographic, clinical, laboratory, surgical and pathological data were obtained retrospectively from electronic health records following the procedure. Tumour staging was based on the 9th edition TNM classification system of the International Association for the Study of Lung Cancer [22]. Diagnoses of diabetes and nonalcoholic fatty liver disease (NAFLD) were identified from self‐reported history or physician documentation. All data were managed and curated using a standardized electronic database to ensure integrity and consistency for analysis.

### 2.3 Health‐Related Lifestyles

Structured interviews assessed smoking status, alcohol use and green tea intake. Smoking was classified as current (within 6 months pre‐enrollment), former (quit ≥ 6 months) or never; alcohol use as current, former or never; regular green tea consumption was defined as ≥ 1 serving/week for ≥ 6 months. Physical activity (PA) was evaluated using the validated International Physical Activity Questionnaire‐Short Form (IPAQ‐SF), which has shown acceptable reliability in Chinese populations (Cronbach's α = 0.82) [23]. Participants reported frequency (days/week) and duration (minutes/day, in ≥ 10‐min bouts) of low‐ (3.3 METs), moderate‐ (4.0 METs) and vigorous‐intensity (8.0 METs) activities during the previous week. Total weekly energy expenditure was calculated as MET‐minutes/week = $\sum \left(\mathrm{MET}\;\text{value}\times \text{duration}\;\left[\min\right]\times \text{frequency}\right)$. PA levels were categorized as high (≥ 3 days vigorous activity totalling ≥ 1500 MET‐min/week or ≥ 7 days of any activity totalling ≥ 3000 MET‐min/week), moderate (≥ 3 days vigorous activity ≥ 20 min/day, ≥ 5 days moderate activity ≥ 30 min/day or ≥ 5 days totalling ≥ 600 MET‐min/week) or low (not meeting moderate or high criteria) [23].

### 2.4 Nutritional Assessment

Nutritional status was evaluated using the modified Patient‐Generated Subjective Global Assessment (mPG‐SGA), a validated tool for oncology populations [24]. The mPG‐SGA consists of two sections: the first mirrors the original PG‐SGA [25], assessing weight loss, dietary intake, symptoms and functional capacity; the second includes only age, scored as 1 if > 65 years, otherwise 0. Total scores classified patients as Stage A (0–2, well‐nourished/mild malnutrition), Stage B (3–6, moderate malnutrition) or Stage C (≥ 7, severe malnutrition) [24].

### 2.5 Anthropometric and Function Measurements

Comprehensive anthropometric and functional assessments were performed, including height, weight, calf circumference (CC), handgrip strength (HGS) and the five‐time chair stand test (CST). Body weight was measured using a digital scale, whereas height was measured using a standardized technique utilizing a 200‐cm metal tape measure with 1‐mm accuracy. The body mass index (BMI), calculated as weight divided by height squared (kg/m^2^), was categorized according to Chinese recommendations as underweight (< 18.5), normal (18.5–23.9), overweight (24.0–27.9) and obese (≥ 28) [26]. CC was measured at the maximal circumference of the left calf using a non‐stretch tape.

HGS was assessed using a calibrated digital dynamometer (CAMRY EH101, China) in accordance with standardized methods [27]. Testing was performed on the dominant hand unless the participant reported recent pain or had undergone surgery (e.g., fusion, arthroplasty, tendon repair and synovectomy) on the wrist or hand of the dominant side within the preceding 3 months. Participants were allowed one practice trial to familiarize themselves with the device. This was followed by two maximal effort trials, separated by a 10‐s rest interval, during which strong verbal encouragement was provided. The higher of the two readings (in kg) was recorded for analysis.

The CST was performed using a standardized 45‐cm height straight‐backed chair without armrests. Participants were instructed to fold their arms across their chest and, upon instruction, to stand up fully and sit down again five times as quickly as possible [28]. The time (in seconds) was measured from the initial movement command until the moment the participant's back touched the chair upon completion of the fifth stand.

### 2.6 Multiparameter BC Analysis

To delineate BC alterations in early‐stage NSCLC patients, multifrequency BIA (InBody 770, InBody Co. Ltd., Seoul, Korea) was employed. Following a standardized protocol, participants fasted for 12 h and refrained from strenuous exercise for 8 h prior to the test. After voiding, removing metal objects and a 10‐min standing equilibration in a thermoneutral environment (22°C–25°C), a rapid BIA measurement was completed within 3–5 min. The system provided a comprehensive BC profile by quantifying 25 variables across the following domains: hydration status (e.g., total body water [TBW] and ECW/ICW), adiposity (e.g., PBF, body fat mass [BFM], visceral fat area [VFA], fat mass index [FMI] and waist–hip ratio [WHR]), lean and muscle composition (e.g., FFM, appendicular skeletal muscle mass [ASM], soft lean mass [SLM] and SMI), cellular health (e.g., body cell mass [BCM] and phase angle [PhA]), protein and mineral content and metabolic indicators [17].

### 2.7 Sarcopenia Assessment

Sarcopenia was diagnosed based on AWGS 2019 criteria, defined as low muscle mass (SMI < 7.0 kg/m^2^ in male and < 5.7 kg/m^2^ in female) plus either low muscle strength (HGS < 28 kg in male and < 18 kg in female) or poor physical performance (CST > 12 s) [12]. Severe sarcopenia required concurrent presence of low muscle mass, low strength and impaired performance. Given the limited sample sizes for sarcopenia (*n* = 27) and severe sarcopenia (*n* = 20) subgroups, primary analyses combined these cases to enhance statistical power.

### 2.8 Statistical Analysis

Normality was assessed using Kolmogorov–Smirnov tests. Continuous variables are expressed as mean ± standard deviation (SD) or median (interquartile range, IQR) and categorical variables as frequencies (%). Group comparisons employed *t*‐tests, Mann–Whitney *U* tests, χ^2^ tests or Fisher's exact tests, depending on data characteristics. Correlations between BC, anthropometric and functional parameters were examined using Spearman's rank correlation coefficient (ρ). A correlation coefficient > 0.7 was considered strong, between 0.3 and 0.7 moderate and below 0.3 weak [29].

Propensity score‐matched (PSM) was performed to minimize confounding via logistic regression with nine covariates: age group, sex, height, diabetes, PA level, mPG‐SGA category, clinical stage, histology and resection extent. Nearest‐neighbour matching with a calliper width of 0.2 and without replacement achieved a 1:4 case‐control match. Balance was assessed using standardized mean differences (SMD < 0.1 considered acceptable) [30].

To identify independent BC factors associated with sarcopenia, multivariable logistic regression was performed using three sequential models: Model 1 (unadjusted), Model 2 (adjusted for age, sex and height) and Model 3 (Model 2 plus PA, diabetes, NAFLD, histology, stage and mPG‐SGA). Results are reported as odds ratios (OR) with 95% confidence intervals (CI). Subgroup analyses stratified by sex (male/female), age (< 60/≥ 60 years), and nutritional status (well‐nourished or mild malnutrition/moderate or severe malnutrition) were conducted, with interaction tested via likelihood ratio tests. Sensitivity analyses further stratified sarcopenic patients into sarcopenia and severe sarcopenia subgroups. All analyses used α = 0.05 and were performed in R v4.2.3 (R Foundation).

## 3 Results

### 3.1 Patient Characteristics and Surgical Outcomes

**FIGURE 1:**
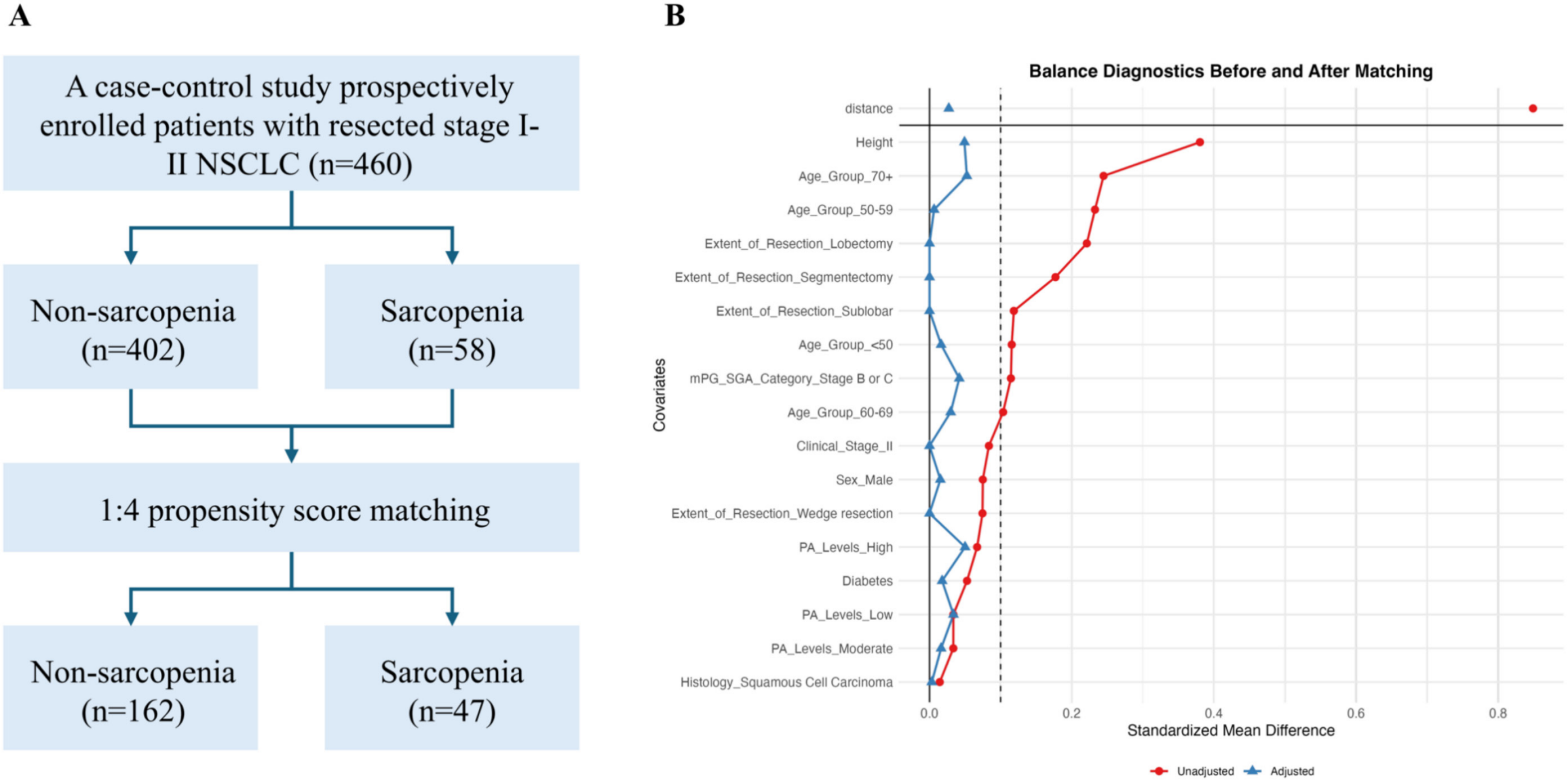
Propensity score matching flowchart and balance diagnostics in patients with resected Stage I–II NSCLC. (A) Flow diagram of patient inclusion and matching. (B) Standardized mean differences (SMD) for covariates before and after matching.

A total of 460 patients with early‐stage NSCLC were initially included in the study. The overall prevalence of sarcopenia in this cohort was 12.61% (58/460). To rigorously control for potential confounders and investigate the specific impact of sarcopenia on BC, we employed a 1:4 PSM design. Following PSM, 47 sarcopenic patients were successfully matched with 162 nonsarcopenic patients, forming the final analytical cohort of 209 subjects. All covariates achieved adequate balance after matching, with SMD below 0.1 (Figure 1 and Table S1). Key demographic and clinical differences observed before matching (e.g., age, education level, smoking history, clinical stage, extent of resection, height and mPG‐SGA category) were no longer statistically significant (all *p* > 0.05; Table 1).

**TABLE 1:** Patient characteristics before and after propensity score matching.

Variables	Before propensity score matching (*n* = 460)			After propensity score matching (*n* = 209)		
	Nonsarcopenia (*n* = 402)	Sarcopenia (*n* = 58)	*p*	Nonsarcopenia (*n* = 162)	Sarcopenia (*n* = 47)	*p*
**Demographic and clinical characteristics**						
Age, years	58.00 [51.00, 65.00]	67.00 [59.50, 73.00]	< 0.001^c^	62.00 [53.25, 69.00]	64.00 [59.00, 72.00]	0.167^c^
Age group (*n*, %)			< 0.001^a^			0.426^a^
< 50 years	95 (23.6)	7 (12.1)		32 (19.8)	7 (14.9)	
50–59 years	149 (37.1)	8 (13.8)		35 (21.6)	8 (17.0)	
60–69 years	97 (24.1)	20 (34.5)		59 (36.4)	16 (34.0)	
70+ years	61 (15.2)	23 (39.7)		36 (22.2)	16 (34.0)	
Sex (%)			0.316^a^			0.741^a^
Female	245 (60.9)	31 (53.4)		88 (54.3)	27 (57.4)	
Male	157 (39.1)	27 (46.6)		74 (45.7)	20 (42.6)	
Education level (*n*, %)			0.009^a^			0.080^a^
Illiterate or Primary School	181 (45.0)	33 (56.9)		79 (48.8)	24 (51.1)	
Junior High School	104 (25.9)	13 (22.4)		39 (24.1)	12 (25.5)	
Senior High School	45 (11.2)	10 (17.2)		17 (10.5)	9 (19.1)	
College or higher	72 (17.9)	2 (3.4)		27 (16.7)	2 (4.3)	
Marital status (*n*, %)			0.007^a^			0.010^b^
Married	389 (96.8)	51 (87.9)		158 (97.5)	41 (87.2)	
Single, divorced or widowed	13 (3.2)	7 (12.1)		4 (2.5)	6 (12.8)	
Residence (*n*, %)			0.178^a^			0.151^a^
Municipalities	146 (36.3)	14 (24.1)		58 (35.8)	12 (25.5)	
Rural	170 (42.3)	29 (50.0)		78 (48.1)	22 (46.8)	
Townships	86 (21.4)	15 (25.9)		26 (16.0)	13 (27.7)	
Smoking history (*n*, %)			0.030^a^			0.875^a^
Current smoker	43 (10.7)	13 (22.4)		24 (14.8)	6 (12.8)	
Former smoker	59 (14.7)	10 (17.2)		26 (16.0)	9 (19.1)	
Non‐smoker	300 (74.6)	35 (60.3)		112 (69.1)	32 (68.1)	
Alcohol consumption status (*n*, %)			0.471^a^			0.442^a^
Current drinker	72 (17.9)	13 (22.4)		40 (24.7)	8 (17.0)	
Former drinker	55 (13.7)	5 (8.6)		22 (13.6)	5 (10.6)	
Non‐drinker	275 (68.4)	40 (69.0)		100 (61.7)	34 (72.3)	
Green tea drinking (*n*, %)			0.325^a^			0.099^a^
No	204 (50.7)	34 (58.6)		77 (47.5)	29 (61.7)	
Yes	198 (49.3)	24 (41.4)		85 (52.5)	18 (38.3)	
Diabetes (*n*, %)	42 (10.4)	3 (5.2)	0.246^a^	14 (8.6)	3 (6.4)	0.768^b^
NAFLD (*n*, %)	48 (11.9)	2 (3.4)	0.068^a^	16 (9.9)	2 (4.3)	0.375^b^
Sedentary time, min	240.00 [120.00, 360.00]	240.00 [120.00, 300.00]	0.155^d^	240.00 [120.00, 300.00]	240.00 [120.00, 300.00]	0.705^d^
Physical activity level (*n*, %)			0.659^a^			1.000^a^
Low	62 (15.4)	7 (12.1)		20 (12.3)	6 (12.8)	
Moderate	159 (39.6)	21 (36.2)		59 (36.4)	17 (36.2)	
High	181 (45.0)	30 (51.7)		83 (51.2)	24 (51.1)	
Histology (*n*, %)			0.485^a^			0.656^b^
Adenocarcinoma	387 (96.3)	55 (94.8)		157 (96.9)	45 (95.7)	
Squamous cell carcinoma	15 (3.7)	3 (5.2)		5 (3.1)	2 (4.3)	
Clinical stage (*n*, %)			0.004^a^			0.400^b^
I	394 (98.0)	52 (89.7)		161 (99.4)	46 (97.9)	
II	8 (2.0)	6 (10.3)		1 (0.6)	1 (2.1)	
Side of pneumonectomy (*n*, %)			0.674^a^			0.500^a^
Left	179 (44.5)	24 (41.4)		60 (37.0)	20 (42.6)	
Right	223 (55.5)	34 (58.6)		102 (63.0)	27 (57.4)	
Extent of resection (*n*, %)			< 0.001^a^			0.980^b^
Lobectomy	119 (29.6)	30 (51.7)		77 (47.5)	22 (46.8)	
Segmentectomy	92 (22.9)	3 (5.2)		11 (6.8)	3 (6.4)	
Sublobar	117 (29.1)	10 (17.2)		34 (21.0)	9 (19.1)	
Wedge resection	74 (18.4)	15 (25.9)		40 (24.7)	13 (27.7)	
**Laboratory parameters**						
Haemoglobin, g/L	138.00 [128.00, 145.75]	132.50 [121.25, 142.75]	0.036^c^	138.00 [128.00, 148.00]	132.00 [122.00, 141.50]	0.048^c^
Prealbumin, g/L	268.00 [234.25, 316.00]	230.00 [200.50, 267.50]	< 0.001^c^	271.50 [229.25, 318.00]	230.00 [201.00, 270.50]	< 0.001^c^
Albumin, g/L	45.20 [43.40, 47.00]	43.50 [41.73, 44.88]	< 0.001^c^	44.90 [42.62, 46.80]	44.10 [41.90, 45.90]	0.162^c^
Globulin, g/L	26.50 [24.40, 28.80]	26.45 [23.83, 28.10]	0.292^c^	26.50 [24.20, 29.20]	26.30 [23.75, 28.15]	0.290^c^
Absolute neutrophil count, ×10^9^/L	3.38 [2.69, 4.29]	3.31 [2.58, 4.07]	0.792^c^	3.49 [2.69, 4.38]	3.19 [2.42, 4.02]	0.186^c^
Lymphocyte count, ×10^9^/L	1.79 [1.46, 2.18]	1.60 [1.21, 2.06]	0.047^c^	1.79 [1.49, 2.25]	1.54 [1.18, 2.04]	0.028^c^
**Surgical outcomes**						
Length of hospital stay, day	6.00 [5.00, 8.00]	9.00 [7.00, 13.00]	< 0.001^c^	7.00 [5.00, 9.00]	9.00 [6.50, 13.00]	0.004^c^
Chest tube drainage time, day	3.50 [3.00, 5.00]	4.00 [3.00, 6.00]	0.003^c^	5.00 [4.00, 6.00]	5.00 [4.00, 6.50]	0.669^c^
Drainage volume in total, mL	397.50 [180.00, 698.75]	467.50 [222.50, 891.25]	0.143^c^	465.00 [212.50, 938.75]	450.00 [205.00, 932.50]	0.927^c^
**Anthropometric parameters**						
Height, m	1.62 [1.58, 1.68]	1.62 [1.54, 1.66]	0.020^d^	1.62 [1.56, 1.67]	1.62 [1.54, 1.65]	0.334^d^
Weight, kg	63.85 [57.10, 70.07]	49.25 [45.12, 55.75]	< 0.001^d^	64.05 [57.05, 72.25]	50.00 [44.95, 56.05]	< 0.001^d^
BMI category (*n*, %)			< 0.001^a^			< 0.001^b^
< 18.5	8 (2.0)	16 (27.6)		2 (1.2)	13 (27.7)	
18.5–24.9	250 (62.2)	41 (70.7)		87 (53.7)	33 (70.2)	
> 24.9	144 (35.8)	1 (1.7)		73 (45.1)	1 (2.1)	
mPG‐SGA category (*n*, %)			0.026^a^			0.490^a^
Well‐nourished or mild malnutrition	351 (87.3)	44 (75.9)		139 (85.8)	38 (80.9)	
Moderate or severe malnutrition	51 (12.7)	14 (24.1)		23 (14.2)	9 (19.1)	
Calf circumference, cm	34.50 [32.50, 36.48]	30.00 [28.92, 32.50]	< 0.001^c^	34.10 [32.50, 36.77]	30.00 [28.95, 33.15]	< 0.001^c^
Handgrip strength/m^2^	10.24 [8.34, 11.98]	8.93 [7.44, 11.12]	0.002^c^	10.22 [8.58, 12.44]	8.81 [7.52, 11.06]	0.002^c^
Chair stand test, sec	11.92 [9.70, 14.85]	14.93 [13.49, 18.03]	< 0.001^c^	12.40 [10.03, 15.30]	14.80 [13.60, 18.20]	< 0.001^c^
**Body composition parameters**						
Waist–hip ratio	0.90 [0.86, 0.95]	0.88 [0.84, 0.93]	0.017^c^	0.91 [0.87, 0.95]	0.88 [0.84, 0.93]	0.047^c^
ASM, kg	18.02 [15.48, 21.41]	14.50 [12.76, 18.13]	< 0.001^c^	18.20 [15.12, 21.67]	14.51 [12.91, 18.26]	< 0.001^c^
Skeletal muscle index, kg/m^2^	6.90 [6.20, 7.50]	5.60 [5.40, 6.68]	< 0.001^c^	7.00 [6.20, 7.68]	5.60 [5.40, 6.80]	< 0.001^c^
Body fat mass, kg	18.05 [14.03, 22.50]	11.85 [8.93, 15.73]	< 0.001^c^	18.60 [14.90, 23.60]	12.70 [9.75, 15.85]	< 0.001^c^
Fat free mass, kg	44.30 [39.62, 51.20]	36.90 [34.18, 43.90]	< 0.001^c^	44.95 [39.10, 51.70]	37.30 [34.00, 44.75]	< 0.001^c^
FFM of trunk, kg	20.30 [18.10, 23.28]	16.75 [15.07, 20.78]	< 0.001^c^	20.70 [18.00, 23.50]	16.70 [15.15, 20.95]	< 0.001^c^
FFM of right arm, kg	2.38 [2.02, 2.90]	1.79 [1.57, 2.46]	< 0.001^c^	2.45 [2.00, 2.96]	1.76 [1.55, 2.54]	< 0.001^c^
FFM of left arm, kg	2.35 [1.99, 2.81]	1.80 [1.52, 2.42]	< 0.001^c^	2.44 [2.01, 2.83]	1.79 [1.48, 2.45]	< 0.001^c^
FFM of right leg, kg	6.69 [5.77, 7.87]	5.54 [4.84, 6.80]	< 0.001^c^	6.75 [5.67, 7.94]	5.56 [4.84, 6.86]	< 0.001^c^
FFM of left leg, kg	6.67 [5.78, 7.88]	5.54 [4.84, 6.73]	< 0.001^c^	6.69 [5.60, 7.91]	5.54 [4.91, 6.88]	< 0.001^c^
Soft lean mass, kg	41.75 [37.30, 48.30]	34.90 [32.25, 41.57]	< 0.001^c^	42.45 [36.80, 49.00]	35.10 [32.05, 42.25]	< 0.001^c^
Visceral fat area, cm^2^	83.95 [64.60, 108.65]	52.35 [43.78, 75.98]	< 0.001^c^	86.65 [67.00, 115.35]	53.90 [45.70, 77.15]	< 0.001^c^
Percent body fat, %	29.00 [23.02, 33.70]	23.45 [19.65, 27.40]	< 0.001^c^	29.80 [24.02, 34.40]	23.60 [21.40, 28.15]	< 0.001^c^
Fat free mass index, kg/m^2^	17.00 [15.90, 18.30]	15.35 [14.20, 16.58]	< 0.001^c^	17.15 [16.10, 18.48]	14.80 [14.10, 16.70]	< 0.001^c^
Fat mass index, kg/m^2^	6.70 [5.20, 8.50]	4.75 [3.80, 6.50]	< 0.001^c^	7.45 [5.45, 8.70]	4.90 [4.00, 6.65]	< 0.001^c^
Phase angle, °	5.30 [4.80, 5.80]	5.00 [4.50, 5.57]	0.003^c^	5.30 [4.80, 5.90]	5.00 [4.55, 5.60]	0.046^c^
Body cell mass, kg	28.75 [25.60, 33.10]	23.85 [22.20, 28.85]	< 0.001^c^	29.10 [25.30, 33.70]	23.90 [22.20, 28.95]	< 0.001^c^
Bone mineral content, kg	2.51 [2.28, 2.81]	2.21 [1.99, 2.59]	< 0.001^c^	2.52 [2.22, 2.79]	2.22 [2.02, 2.64]	0.002^c^
Protein, kg	8.70 [7.70, 10.00]	7.25 [6.70, 8.90]	< 0.001^c^	8.80 [7.60, 10.20]	7.30 [6.70, 8.90]	< 0.001^c^
Minerals, kg	3.04 [2.77, 3.41]	2.71 [2.40, 3.16]	< 0.001^c^	3.05 [2.69, 3.43]	2.74 [2.41, 3.20]	0.002^c^
Total body water, kg	32.60 [29.20, 37.70]	27.35 [25.05, 32.80]	< 0.001^c^	33.05 [28.72, 38.38]	27.40 [24.90, 33.00]	< 0.001^c^
Extracellular water, kg	12.55 [11.30, 14.50]	10.75 [9.80, 13.05]	< 0.001^c^	12.80 [11.10, 14.78]	10.80 [9.50, 13.20]	< 0.001^c^
Intracellular water, kg	20.05 [17.90, 23.10]	16.55 [15.35, 20.20]	< 0.001^c^	20.30 [17.70, 23.50]	16.60 [15.30, 20.35]	< 0.001^c^
ECW/ICW, %	0.63 [0.61, 0.64]	0.64 [0.63, 0.66]	< 0.001^c^	0.63 [0.62, 0.64]	0.64 [0.63, 0.66]	< 0.001^c^
Basal metabolic rate, kcal	1328.00 [1227.00, 1475.00]	1150.50 [1107.75, 1318.50]	< 0.001^c^	1340.50 [1214.00, 1486.75]	1151.00 [1104.00, 1336.50]	< 0.001^c^

Abbreviations: ASM, appendicular skeletal muscle mass; BMI, body mass index; ECW, extracellular water; FFM, fat free mass; ICW, intracellular water; mPG‐SGA, modified patient‐generated subjective global assessment; NAFLD, non‐alcoholic fatty liver disease.

^a^ Chi‐square test.

^b^ Fisher's exact test.

^c^ Mann–Whiney *U* test.

^d^ Two‐sample *t*‐test.

Patients with sarcopenia exhibited significantly lower body weight (50.00 vs. 64.05 kg, *p* < 0.001), reduced CC (30.00 vs. 34.10 cm, *p* < 0.001), diminished height‐adjusted HGS (HGS/m^2^, 8.81 vs. 10.22 kg/m^2^, *p* = 0.002) and prolonged CST duration (14.80 vs. 12.40 s, *p* < 0.001). The proportion of underweight individuals was markedly higher in the sarcopenic group (27.7% vs. 1.2%), though one sarcopenic patient was classified as obese. Laboratory findings indicated significantly lower haemoglobin (132.00 vs. 138.00 g/L, *p* = 0.048) and prealbumin (230.00 vs. 271.50 g/L, *p* < 0.001) in sarcopenic patients. Regarding surgical outcomes, this group experienced longer total hospital stays (9.00 vs. 7.00 days, *p* < 0.001).

### 3.2 BC Differences

BIA revealed profound systemic alterations in sarcopenic patients that extended beyond mere reductions in muscle mass (Table 1). These individuals exhibited significant depletion of FFM across all anatomical compartments (trunk: 16.70 vs. 20.70 kg; right arm: 1.76 vs. 2.45 kg; left arm: 1.79 vs. 2.44 kg; right leg: 5.56 vs. 6.75 kg; left leg: 5.54 vs. 6.69 kg; all *p* < 0.001). Compromised cellular health was also observed, reflected in lower PhA (5.00° vs. 5.30°, *p* = 0.046), elevated ECW/ICW ratio (0.64 vs. 0.63, *p* < 0.001) and decreased BCM (23.90 vs. 29.10 kg, *p* < 0.001). Concurrent metabolic dysregulation was evidenced by reduced BMR (1151.00 vs. 1340.50 kcal, *p* < 0.001), protein content (7.30 vs. 8.80 kg, *p* < 0.001), mineral mass (2.74 vs. 3.05 kg, *p* = 0.002) and bone mineral content (BMC, 2.22 vs. 2.52 kg, *p* = 0.002). Adipose‐related metrics were also markedly disrupted, including VFA (53.90 vs. 86.65 cm^2^, *p* < 0.001), BFM (12.70 vs. 18.60 kg, *p* < 0.001), PBF (23.60% vs. 29.80%, *p* < 0.001) and WHR (0.88 vs. 0.91, *p* = 0.047).

### 3.3 Sex‐Stratified BC Profiling

Distinct sex‐specific patterns of BC alteration were identified in sarcopenic patients (Table 2). Sarcopenic females exhibited systemic depletion affecting not only muscle but also adipose and mineral compartments: ASM (13.10 vs. 15.43 kg, *p* < 0.001) and SMI (5.40 vs. 6.30 kg/m^2^, *p* < 0.001) were significantly reduced, along with adipose metrics including BFM (11.20 vs. 19.20 kg, *p* < 0.001), VFA (50.30 vs. 95.15 cm^2^, *p* < 0.001), PBF (25.41% vs. 32.88%, *p* < 0.001) and FMI (4.80 vs. 8.00 kg/m^2^, *p* < 0.001). Mineral mass (2.51 vs. 2.76 kg, *p* < 0.001) and BMC (2.05 vs. 2.26 kg, *p* < 0.001) were also significantly lower.

**TABLE 2:** Basic characteristics and body composition parameters in patients with early‐stage resectable NSCLC, according to sex.

Variables	Female (*n* = 95)			Male (*n* = 83)		
	Nonsarcopenia (*n* = 88)	Sarcopenia (*n* = 27)	*p*	Nonsarcopenia (*n* = 66)	Sarcopenia (*n* = 17)	*p*
**Demographic and clinical characteristics**						
Age, years	60.00 [49.75, 69.00]	61.00 [52.50, 71.00]	0.452^c^	63.00 [58.25, 71.00]	67.00 [61.75, 73.25]	0.157^c^
Age group (*n*, %)			0.615^a^			0.695^b^
< 50 years	23 (26.1)	6 (22.2)		9 (12.2)	1 (5.0)	
50–59years	22 (25.0)	5 (18.5)		13 (17.6)	3 (15.0)	
60–69years	27 (30.7)	8 (29.6)		32 (43.2)	8 (40.0)	
70+ years	16 (18.2)	8 (29.6)		20 (27.0)	8 (40.0)	
Education level (*n*, %)			0.483^b^			0.164^b^
Illiterate or Primary School	51 (58.0)	17 (63.0)		28 (37.8)	7 (35.0)	
Junior High School	15 (17.0)	4 (14.8)		24 (32.4)	8 (40.0)	
Senior High School	7 (8.0)	4 (14.8)		10 (13.5)	5 (25.0)	
College or higher	15 (17.0)	2 (7.4)		12 (16.2)	0 (0.0)	
Marital status (*n*, %)			0.086^b^			0.043^b^
Married	84 (95.5)	23 (85.2)		74 (100.0)	18 (90.0)	
Single, divorced or widowed	4 (4.5)	4 (14.8)		0 (0.0)	2 (10.0)	
Residence (*n*, %)			0.155^b^			0.715^b^
Municipalities	32 (36.4)	6 (22.2)		26 (35.1)	6 (30.0)	
Rural	43 (48.9)	13 (48.1)		35 (47.3)	9 (45.0)	
Townships	13 (14.8)	8 (29.6)		13 (17.6)	5 (25.0)	
Smoking history (*n*, %)			0.807^b^			0.503^a^
Current smoker	2 (2.3)	1 (3.7)		22 (29.7)	5 (25.0)	
Former smoker	3 (3.4)	0 (0.0)		23 (31.1)	9 (45.0)	
Non‐smoker	83 (94.3)	26 (96.3)		29 (39.2)	6 (30.0)	
Alcohol consumption status (*n*, %)			0.493^b^			0.524^b^
Current drinker	8 (9.1)	2 (7.4)		32 (43.2)	6 (30.0)	
Former drinker	6 (6.8)	0 (0.0)		16 (21.6)	5 (25.0)	
Non‐drinker	74 (84.1)	25 (92.6)		26 (35.1)	9 (45.0)	
Green tea drinking (*n*, %)			0.182^a^			0.628^a^
No	54 (61.4)	21 (77.8)		23 (31.1)	8 (40.0)	
Yes	34 (38.6)	6 (22.2)		51 (68.9)	12 (60.0)	
Diabetes (*n*, %)	5 (5.7)	1 (3.7)	1.000^b^	9 (12.2)	2 (10.0)	1.000^b^
NAFLD (*n*, %)	7 (8.0)	1 (3.7)	0.678^b^	9 (12.2)	1 (5.0)	0.683^b^
Sedentary time, min	180.00 [120.00, 300.00]	240.00 [180.00, 300.00]	0.694^c^	240.00 [120.00, 300.00]	240.00 [60.00, 300.00]	0.378^c^
Physical activity level (*n*, %)			1.000^b^			0.943^b^
Low	10 (11.4)	3 (11.1)		10 (13.5)	3 (15.0)	
Moderate	28 (31.8)	8 (29.6)		31 (41.9)	9 (45.0)	
High	50 (56.8)	16 (59.3)		33 (44.6)	8 (40.0)	
Histology (*n*, %)			NA			0.638^b^
Adenocarcinoma	88 (100.0)	27 (100.0)		69 (93.2)	18 (90.0)	
Squamous cell carcinoma	0 (0.0)	0 (0.0)		5 (6.8)	2 (10.0)	
Clinical stage (%)			0.416^b^			NA
I	87 (98.9)	26 (96.3)		74 (100.0)	20 (100.0)	
II	1 (1.1)	1 (3.7)		0 (0.0)	0 (0.0)	
Side of pneumonectomy (*n*, %)			0.383^a^			1.000^a^
Left	32 (36.4)	13 (48.1)		28 (37.8)	7 (35.0)	
Right	56 (63.6)	14 (51.9)		46 (62.2)	13 (65.0)	
Extent of resection (*n*, %)			0.711^b^			0.641^b^
Lobectomy	41 (46.6)	10 (37.0)		36 (48.6)	12 (60.0)	
Segmentectomy	6 (6.8)	2 (7.4)		5 (6.8)	1 (5.0)	
Sublobar	13 (14.8)	6 (22.2)		21 (28.4)	3 (15.0)	
Wedge resection	28 (31.8)	9 (33.3)		12 (16.2)	4 (20.0)	
**Laboratory parameters**						
Haemoglobin, g/L	131.50 [125.00, 139.00]	127.00 [119.00, 136.50]	0.071^c^	144.50 [139.25, 153.00]	141.50 [132.75, 150.50]	0.307^c^
Prealbumin, g/L	254.50 [222.75, 288.25]	215.00 [173.50, 252.50]	0.001^c^	284.50 [261.25, 356.50]	265.00 [220.25, 296.25]	0.037^c^
Albumin, g/L	44.90 [42.70, 46.82]	44.10 [42.40, 46.85]	0.588^c^	44.85 [42.60, 46.77]	43.45 [41.58, 44.82]	0.119^c^
Globulin, g/L	27.50 [25.08, 29.85]	26.90 [24.90, 28.15]	0.047^c^	25.25 [23.02, 27.80]	25.60 [23.67, 28.27]	0.651^c^
Absolute neutrophil count, ×10^9^/L	3.08 [2.35, 4.08]	2.91 [2.28, 3.80]	0.582^c^	3.90 [3.12, 4.79]	3.42 [3.09, 4.07]	0.250^c^
Lymphocyte count, ×10^9^/L	1.81 [1.51, 2.25]	1.53 [1.17, 1.95]	0.028^c^	1.79 [1.45, 2.20]	1.64 [1.19, 2.04]	0.383^c^
**Surgical outcomes**						
Length of hospital stay, day	7.00 [5.00, 8.00]	8.00 [5.50, 12.00]	0.029^c^	7.00 [6.00, 10.00]	9.00 [7.00, 13.00]	0.062^c^
Chest tube drainage time, day	4.00 [3.00, 5.00]	4.00 [3.00, 5.50]	0.726^c^	4.00 [3.00, 6.00]	5.00 [4.00, 6.25]	0.182^c^
Drainage volume, mL	425.00 [197.50, 701.25]	340.00 [205.00, 592.50]	0.661^c^	590.00 [312.50, 1165.00]	737.50 [238.75, 1498.75]	0.589^c^
**Anthropometric parameters**						
Height, m	1.57 [1.53, 1.60]	1.57 [1.52, 1.62]	0.955^c^	1.68 [1.65, 1.72]	1.67 [1.64, 1.69]	0.138^c^
Weight, kg	59.60 [53.72, 67.25]	46.30 [43.30, 49.50]	< 0.001^c^	68.70 [63.85, 75.28]	56.60 [53.70, 61.98]	< 0.001^c^
BMI category (*n*, %)			< 0.001^b^			< 0.001^b^
< 18.5	1 (1.1)	12 (44.4)		1 (1.4)	1 (5.0)	
18.5–24.9	49 (55.7)	14 (51.9)		38 (51.4)	19 (95.0)	
> 24.9	38 (43.2)	1 (3.7)		35 (47.3)	0 (0.0)	
mPG‐SGA category (*n*, %)			0.385^a^			1.000^b^
Well‐nourished or mild Malnutrition	71 (80.7)	19 (70.4)		68 (91.9)	19 (95.0)	
Moderate or severe malnutrition	17 (19.3)	8 (29.6)		6 (8.1)	1 (5.0)	
Calf circumference, cm	34.00 [32.10, 36.42]	29.80 [28.00, 32.00]	< 0.001^c^	34.50 [33.05, 37.38]	31.10 [29.58, 34.00]	< 0.001^c^
Handgrip strength/m^2^	9.22 [8.19, 10.25]	7.70 [6.88, 8.93]	0.001^c^	12.45 [10.49, 13.72]	11.28 [9.12, 11.91]	0.033^c^
Chair stand test, sec	12.79 [10.02, 15.06]	14.80 [13.39, 18.63]	< 0.001^c^	12.30 [10.14, 15.56]	15.06 [13.91, 18.02]	0.001^c^
**Body composition parameters**						
Waist–hip ratio	0.91 [0.87, 0.96]	0.85 [0.83, 0.89]	< 0.001^c^	0.91 [0.87, 0.94]	0.92 [0.89, 1.43]	0.155^c^
ASM, kg	15.43 [14.35, 16.96]	13.10 [11.78, 14.46]	< 0.001^c^	21.72 [20.45, 23.75]	18.92 [17.41, 19.57]	< 0.001^c^
Skeletal muscle index, kg/m^2^	6.30 [6.00, 6.80]	5.40 [5.20, 5.50]	< 0.001^c^	7.70 [7.30, 8.10]	6.80 [6.52, 6.90]	< 0.001^c^
Body fat mass, kg	19.20 [16.30, 24.33]	11.20 [9.05, 15.95]	< 0.001^c^	17.85 [13.55, 21.73]	13.30 [11.10, 15.57]	0.004^c^
Fat free mass, kg	39.45 [37.40, 42.28]	34.40 [32.70, 36.70]	< 0.001^c^	51.90 [48.58, 55.95]	44.80 [40.35, 46.52]	< 0.001^c^
FFM of trunk, kg	18.15 [17.15, 19.50]	15.60 [14.65, 16.40]	< 0.001^c^	23.75 [22.52, 25.40]	21.10 [19.92, 21.92]	< 0.001^c^
FFM of right arm, kg	2.02 [1.87, 2.28]	1.56 [1.46, 1.70]	< 0.001^c^	2.99 [2.77, 3.21]	2.54 [2.31, 2.63]	< 0.001^c^
FFM of left arm, kg	2.04 [1.87, 2.26]	1.55 [1.39, 1.73]	< 0.001^c^	2.90 [2.66, 3.31]	2.47 [2.28, 2.51]	< 0.001^c^
FFM of right leg, kg	5.76 [5.22, 6.25]	5.04 [4.46, 5.48]	< 0.001^c^	7.97 [7.46, 8.70]	7.04 [6.38, 7.22]	< 0.001^c^
FFM of left leg, kg	5.66 [5.26, 6.28]	4.93 [4.42, 5.47]	< 0.001^c^	7.92 [7.53, 8.61]	7.03 [6.41, 7.23]	< 0.001^c^
Soft lean mass, kg	37.10 [35.20, 39.90]	32.40 [30.75, 34.60]	< 0.001^c^	49.10 [45.92, 52.90]	42.35 [38.22, 43.95]	< 0.001^c^
Visceral fat area, cm^2^	95.15 [71.40, 126.23]	50.30 [41.20, 81.35]	< 0.001^c^	79.20 [64.38, 97.58]	59.20 [49.75, 75.52]	0.004^c^
Percent body fat, %	32.88 ± 6.06	25.41 ± 6.76	< 0.001^d^	24.29 ± 7.12	23.04 ± 4.71	0.463^d^
Fat free mass index, kg/m^2^	16.30 [15.40, 17.02]	14.20 [13.75, 14.55]	< 0.001^c^	18.35 [17.62, 19.58]	16.70 [16.15, 17.05]	< 0.001^c^
Fat mass index, kg/m^2^	8.00 [6.40, 9.80]	4.80 [3.85, 6.70]	< 0.001^c^	6.25 [4.70, 7.97]	5.55 [4.47, 6.58]	0.294^c^
Phase angle, °	5.10 [4.60, 5.60]	4.80 [4.25, 5.25]	0.083^c^	5.60 [5.00, 6.40]	5.15 [4.88, 5.85]	0.322^c^
Body cell mass, kg	25.30 [24.20, 27.42]	22.30 [21.10, 23.60]	< 0.001^c^	33.85 [31.55, 36.27]	29.35 [26.87, 30.05]	< 0.001^c^
Bone mineral content, kg	2.26 [2.14, 2.42]	2.05 [1.90, 2.17]	< 0.001^c^	2.80 [2.65, 3.06]	2.64 [2.42, 3.14]	0.141^c^
Protein, kg	7.60 [7.30, 8.30]	6.80 [6.40, 7.15]	< 0.001^c^	10.25 [9.53, 10.90]	8.95 [8.78, 9.53]	< 0.001^c^
Minerals, kg	2.76 [2.57, 2.93]	2.51 [2.29, 2.63]	< 0.001^c^	3.44 [3.27, 3.72]	3.20 [3.02, 3.68]	0.072^c^
Total body water, kg	28.95 [27.50, 31.15]	25.20 [24.00, 27.05]	< 0.001^c^	38.40 [35.95, 41.38]	33.25 [30.93, 34.35]	< 0.001^c^
Extracellular water, kg	11.30 [10.60, 12.10]	9.70 [9.30, 10.65]	< 0.001^c^	14.85 [14.03, 16.08]	13.20 [12.65, 13.80]	< 0.001^c^
Intracellular water, kg	17.70 [16.90, 19.12]	15.50 [14.65, 16.25]	< 0.001^c^	23.60 [22.02, 25.37]	20.60 [19.82, 21.38]	< 0.001^c^
ECW/ICW, %	0.63 [0.62, 0.64]	0.64 [0.62, 0.66]	0.101^c^	0.63 [0.62, 0.64]	0.65 [0.64, 0.67]	< 0.001^c^
Basal metabolic rate, kcal	1222.50 [1177.75, 1283.25]	1113.00 [1077.00, 1157.50]	< 0.001^c^	1491.50 [1419.50, 1578.25]	1337.00 [1148.50, 1375.50]	< 0.001^c^

Abbreviations: ASM, appendicular skeletal muscle mass; BMI, body mass index; ECW, extracellular water; FFM, fat free mass; ICW, intracellular water; mPG‐SGA, modified patient‐generated subjective global assessment; NAFLD, non‐alcoholic fatty liver disease.

^a^ Chi‐square test.

^b^ Fisher's exact test.

^c^ Mann–Whiney *U* test.

^d^ Two‐sample *t*‐test.

In contrast, sarcopenic males showed selective deficits in muscle mass without significant adipose or mineral loss: ASM (18.92 vs. 21.72 kg, *p* < 0.001), SMI (6.80 vs. 7.70 kg/m^2^, *p* < 0.001) and FFM (44.80 vs. 51.90 kg, *p* < 0.001) were markedly reduced, while adipose parameters (PBF: 23.04% vs. 24.29%, *p* = 0.463; FMI: 5.55 vs. 6.25 kg/m^2^, *p* = 0.294) and mineral content (mineral mass: 3.20 vs. 3.44 kg, *p* = 0.072; BMC: 2.64 vs. 2.80 kg, *p* = 0.141) remained statistically unchanged. Notably, cellular health deterioration was consistent across sexes, with females showing a pronounced reduction in PhA.

### 3.4 Correlation Analysis

**FIGURE 2:**
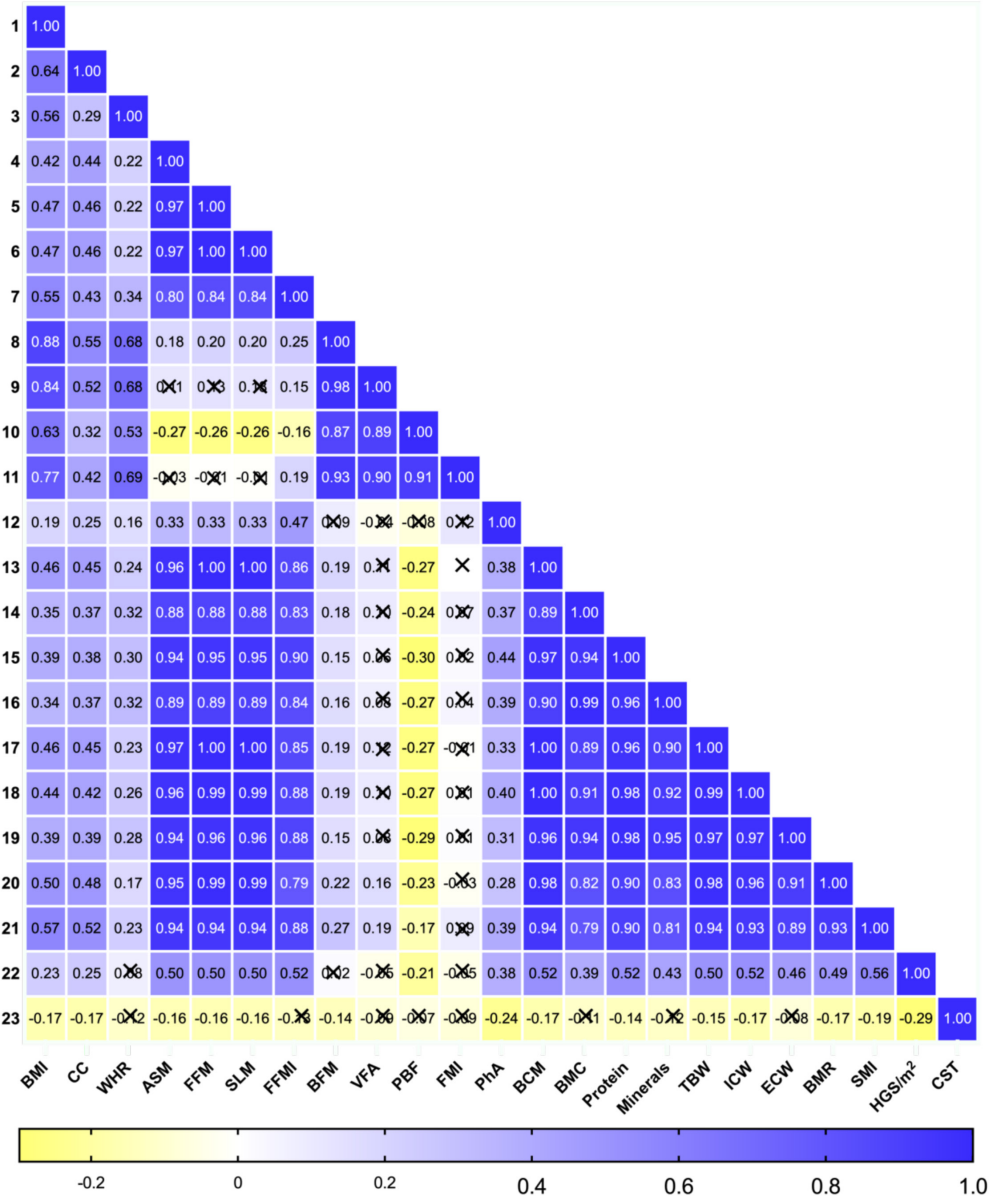
Correlation analysis among the anthropometric parameters and body composition parameters. The horizontal axis indicates the direction and intensity of the correlation coefficient between the two variables intersecting at the single cell. Crosses refer to not significant Spearman's correlation coefficients. Abbreviations: ASM, appendicular skeletal muscle mass; BCM, body cell mass; BFM, body fat mass; BMC, bone mineral content; BMR, Basal metabolic rate; CST, chair stand test; ECW, extracellular water; FFM, fat free mass; FFMI, fat free mass index; FMI, fat mass index; HGS, handgrip strength; ICW, intracellular water; PBF, percent body fat; PhA: phase angle; SLM, soft lean mass; SMI, skeletal muscle index; TBW, total body water; VFA, visceral fat area; WHR, waist–hip ratio.

Spearman correlation analysis revealed significant relationships among anthropometric, functional and BC parameters (Figure 2). SMI showed strong positive correlations with ASM (ρ = 0.94, *p* < 0.001), FFM (ρ = 0.94, *p* < 0.001), SLM (ρ = 0.94, *p* < 0.001), FFMI (ρ = 0.88, *p* < 0.001), BCM (ρ = 0.94, *p* < 0.001), protein content (ρ = 0.90, *p* < 0.001), mineral mass (ρ = 0.81, *p* < 0.001), TBW (ρ = 0.93, *p* < 0.001), ICW (ρ = 0.89, *p* < 0.001) and BMR (ρ = 0.93, *p* < 0.001). PhA was moderately correlated with ASM (ρ = 0.33, *p* < 0.001), FFM (ρ = 0.33, *p* < 0.001), SLM (ρ = 0.33, *p* < 0.001), FFMI (ρ = 0.47, *p* < 0.001), protein content (ρ = 0.44, *p* < 0.001), mineral mass (ρ = 0.39, *p* < 0.001), ICW (ρ = 0.40, *p* < 0.001), SMI (ρ = 0.39, *p* < 0.001), HGS/m^2^ (ρ = 0.38, *p* < 0.001) and correlated negatively with CST time (ρ = −0.24, *p* = 0.044). PBF showed moderate or strong positive correlations with all adiposity indices (BMI: ρ = 0.63; WHR: ρ = 0.53; BFM: ρ = 0.87; VFA: ρ = 0.89; FMI: ρ = 0.91; all *p* < 0.001) but negatively correlated with parameters reflecting hydration status (TBW: ρ = −0.27, *p* = 0.007; ICW: ρ = −0.27, *p* = 0.006; ECW: ρ = −0.29, *p* = 0.001), cellular health (BCM: ρ = −0.27, *p* = 0.008), protein content (ρ = −0.30, *p* = 0.001), mineral mass (BMC: ρ = −0.24, *p* = 0.038), BMR (ρ = −0.23, *p* = 0.048) and HGS/m^2^ (ρ = −0.21, *p* = 0.001).

### 3.5 Multivariable Logistic Regression Analysis

In the fully adjusted model (Model 3), several BC parameters were significantly associated with sarcopenia (Table 3). Higher values of ASM (OR = 0.03, 95%CI: 0.01–0.08), FFM (OR = 0.37, 0.25–0.49), SLM (OR = 0.35, 0.24–0.47), BCM (OR = 0.27, 0.16–0.39), protein content (OR = 0.22, 0.12–0.39), ICW (OR = 0.19, 0.11–0.31) and ECW (OR = 0.22, 0.12–0.35) were associated with a lower likelihood of sarcopenia (all *p* < 0.001). Similarly, higher values of BMI (OR = 0.43, 0.32–0.54), CC (OR = 0.64, 0.54–0.74), BFM (OR = 0.81, 0.75–0.87), PBF (OR = 0.89, 0.85–0.94), FMI (OR = 0.68, 0.56–0.80), VFA (OR = 0.96, 0.95–0.97) and BMR (OR = 0.95, 0.94–0.97; all *p* < 0.001) were also associated with reduced odds of sarcopenia. Conversely, a higher WHR was associated with increased odds of sarcopenia (OR = 3.51, 1.60–19.55, *p* = 0.036). PhA showed no independent association (OR = 1.06, 0.72–1.50, *p* = 0.769).

**TABLE 3:** Association between multiple body composition parameters and sarcopenia.

Variables	Model 1		Model 2		Model 3	
	OR (95%CI)	*p*	OR (95%CI)	*p*	OR (95%CI)	*p*
BMI	0.46 (0.35, 0.56)	< 0.001	0.43 (0.33, 0.54)	< 0.001	0.43 (0.32, 0.54)	< 0.001
Calf circumference	0.65 (0.55, 0.74)	< 0.001	0.64 (0.55, 0.74)	< 0.001	0.64 (0.54, 0.74)	< 0.001
WHR	2.95 (1.46, 16.09)	0.045	3.22 (1.50, 18.20)	0.040	3.51 (1.60, 19.55)	0.036
ASM	0.77 (0.68, 0.85)	< 0.001	0.04 (0.01, 0.10)	< 0.001	0.03 (0.01, 0.08)	< 0.001
FFM	0.85 (0.79, 0.90)	< 0.001	0.43 (0.31, 0.54)	< 0.001	0.37 (0.25, 0.49)	< 0.001
SLM	0.84 (0.79, 0.90)	< 0.001	0.41 (0.28, 0.52)	< 0.001	0.35 (0.24, 0.47)	< 0.001
FFMI	0.57 (0.45, 0.70)	< 0.001	0.36 (0.25, 0.50)	< 0.001	0.33 (0.22, 0.47)	< 0.001
BFM	0.82 (0.76, 0.88)	< 0.001	0.82 (0.75, 0.88)	< 0.001	0.81 (0.75, 0.87)	< 0.001
VFA	0.96 (0.95, 0.98)	< 0.001	0.96 (0.95, 0.98)	< 0.001	0.96 (0.95, 0.97)	< 0.001
PBF	0.92 (0.88, 0.97)	< 0.001	0.90 (0.85, 0.94)	< 0.001	0.89 (0.85, 0.94)	< 0.001
FMI	0.71 (0.60, 0.83)	< 0.001	0.68 (0.57, 0.80)	< 0.001	0.68 (0.56, 0.80)	< 0.001
PhA	0.96 (0.68, 1.29)	0.784	1.00 (0.69, 1.41)	0.986	1.06 (0.72, 1.50)	0.769
BCM	0.79 (0.71, 0.86)	< 0.001	0.31 (0.20, 0.43)	< 0.001	0.27 (0.16, 0.39)	< 0.001
BMC	1.30 (0.93, 1.86)	0.120	1.45 (1.00, 2.32)	0.069	1.53 (1.04, 2.55)	0.051
Protein	0.65 (0.50, 0.82)	0.001	0.24 (0.13, 0.41)	< 0.001	0.22 (0.12, 0.39)	< 0.001
Mineral	1.19 (0.84, 1.65)	0.292	1.32 (0.92, 2.02)	0.141	1.39 (0.95, 2.20)	0.103
TBW	0.81 (0.74, 0.88)	< 0.001	0.34 (0.23, 0.46)	< 0.001	0.29 (0.18, 0.41)	< 0.001
ICW	0.74 (0.64, 0.83)	< 0.001	0.23 (0.14, 0.36)	< 0.001	0.19 (0.11, 0.31)	< 0.001
ECW	0.71 (0.59, 0.84)	< 0.001	0.25 (0.15, 0.39)	< 0.001	0.22 (0.12, 0.35)	< 0.001
BMR	0.99 (0.99, 0.99)	< 0.001	0.96 (0.94, 0.97)	< 0.001	0.95 (0.94, 0.97)	< 0.001

*Note:* Model 1: unadjusted model; Model 2: adjusted with age group, sex and height; Model 3: further adjusted with physical activity level, diabetes, NAFLD, histology, clinical stage and modified patient‐generated subjective global assessment based on Model 2.

Abbreviations: ASM, appendicular skeletal muscle mass; BCM, body cell mass; BFM, body fat mass; BMC, bone mineral content; BMI, body mass index; BMR, basal metabolic rate; ECW, extracellular water; FFM, fat free mass; FFMI, fat free mass index; FMI, fat mass index; ICW, intracellular water; PBF, percent body fat; PhA, phase angle; SLM, soft lean mass; TBW, total body water; VFA, visceral fat area; WHR, waist–hip ratio.

### 3.6 Subgroup and Sensitivity Analyses

Sex significantly modified the association between BC and sarcopenia (Tables S2–S4). For instance, elevated WHR was more strongly associated with sarcopenia in males (OR = 8.80, 2.36–32.83) than in females (OR = 1.66, 0.77–3.56; *p* for interaction = 0.004). In contrast, reductions in SLM (female OR = 0.71, 0.62–0.83; male OR = 0.89, 0.82–0.96; *p* for interaction = 0.007), FFM (female OR = 0.82, 0.75–0.89; male OR = 0.97, 0.91–1.02; *p* for interaction = 0.001) and PBF (female OR = 0.47, 0.34–0.63; male OR = 0.90, 0.74–1.11; *p* for interaction < 0.001) showed stronger inverse associations with sarcopenia in females than in males. No significant interactions were observed for age or nutritional status. Sensitivity analyses further categorizing the cohort into nonsarcopenic, sarcopenic and severe sarcopenic subgroups corroborated the primary findings (Tables S5, S6).

## 4 Discussion

To the best of our knowledge, this study provides the first comprehensive analysis of multidimensional BC alterations, as assessed via BIA, in patients with early‐stage NSCLC and preoperative sarcopenia. The prevalence of sarcopenia in our cohort was 12.6% (58/460), which is comparable to the overall prevalence of 16.8% reported for all‐stage lung cancer patients in a large Chinese national survey [31]. Our primary findings indicate that sarcopenia in this population is not merely a disorder of reduced muscle mass, but rather a systemic catabolic condition characterized by concurrent depletion of adipose tissue, protein and mineral reserves. BIA‐derived BC profiling further revealed significant cellular and metabolic dysfunction, exemplified by elevated ECW/ICW ratios, reduced BCM and decreased BMR. Notably, a fundamental sexual dimorphism was observed: Females exhibited coordinated muscle‐adipose‐mineral loss, whereas males presented with isolated myopenia with preserved adiposity and minerals.

Previous research has extensively explored the relationship between BC and clinical outcomes in cancer, predominantly focusing on lean and adipose tissue quantified by CT. For instance, Sun et al. highlighted the prognostic utility of combining pectoralis muscle mass and density at the fourth thoracic vertebra level [32], while Huang et al. highlighted the divergent roles of different adipose depots (e.g., subcutaneous versus intermuscular adipose tissue) [2]. Complementing these detailed morphological insights from CT; BIA provides physiological and functional status of body tissues, including cellular integrity, metabolic activity and fluid balance [17]. Consistent with a prior BIA survey of community‐dwelling older adults, similar multidimensional deterioration was observed in our preoperative sarcopenic patients [17]. This pattern, which extends beyond simple malnutrition, implies a state of systemic, tumour‐induced metabolic reprogramming. As shown by Agca et al., remote tumour growth induces profound transcriptomic changes in muscle tissue, promoting a shift toward atrophic gene expression (e.g., upregulation of Atrogin‐1 and MuRF1) while suppressing pathways related to oxidative metabolism [33]. The consequent suppression of oxidative phosphorylation provides a plausible mechanism for the reduced BMR and impaired cellular health (e.g., PhA, BCM and ECW/ICW) observed in our cohort. This coordinated degradation extends beyond myonuclei to involve mononuclear cells such as fibro‐adipogenic progenitors, indicating a wholesale remodelling of the muscle microenvironment [33]. Concurrent visceral adipose tissue loss further reflects activated lipolysis within this systemic catabolic state. The intercorrelations among these BC parameters reinforce that cancer‐associated sarcopenia represents a manifestation of a whole‐body metabolic crisis, not merely an isolated muscle disorder. Collectively, BIA provides a holistic physiological perspective that effectively complements the anatomical detail from CT. Coupled with its inherent practical advantages—being non‐invasive, rapid, easy to administer and requiring minimal operator training—BIA emerges not only as a feasible tool for routine clinical use but also as a valuable adjunct to standard preoperative evaluation. Integrating it into workflows for resectable NSCLC would enable a more comprehensive risk assessment and facilitate timely, phenotype‐specific prehabilitation, with the ultimate goal of improving postoperative resilience and clinical outcomes.

A pivotal finding of this study is the pronounced sexual dimorphism in the phenotypic presentation of sarcopenia, spanning systemic BC to cellular‐level physiology (Tables 2 and S2). Sarcopenic females exhibited significant reductions across all adipose metrics, including BFM, PBF, FMI and VFA, compared to nonsarcopenic controls, whereas these parameters remained largely unchanged in sarcopenic males. This divergence was corroborated by a strong, inverse association between PBF and sarcopenia exclusive to females (OR = 0.47, 95% CI: 0.34–0.63), with a significant sex‐interaction effect (*p* for interaction < 0.001). Notably, this sex‐specific pattern was also evident at the cellular level: While PhA lost its independent association with sarcopenia in the combined‐sex cohort, sex‐stratified analysis confirmed a significant link in females but not in males. This suggests that the female sarcopenic phenotype involves more profound systemic cellular dysfunction, and that combining both sexes can obscure critical biological associations.

The observed pattern aligns with emerging metabolic insights. Beckner et al. have suggested that females may possess an enhanced capacity to mobilize and oxidize fat as a preferred energy substrate during systemic stress, a metabolic adaptation that could paradoxically help mitigate lean tissue catabolism [34]. Concurrently, adipose tissue—particularly subcutaneous fat—functions not merely as a passive energy reservoir but as an active endocrine organ [35]. In females, it serves as the primary site for the aromatization of androgens into estrogens [36]. Consequently, the profound loss of adipose tissue in sarcopenic females may disrupt this endocrine function, potentially exacerbating muscle wasting by diminishing local oestrogen production, which is known to exert anabolic and anti‐inflammatory effects on skeletal muscle [36]. This provides a plausible mechanism for the coordinated fat‐muscle loss observed in sarcopenic females as a metabolically driven response to tumour‐induced cachexia. Furthermore, the significant reduction in BMC specifically in sarcopenic females further supports a shared pathophysiology linking oestrogen status to concurrent sarcopenia and osteopenia [37]. In summary, the sex‐specific alterations in adipose tissue, cellular integrity and bone mass strongly indicate that the pathophysiology of cancer‐associated sarcopenia is distinct between sexes. These findings not only warrant further mechanistic investigation but also carry direct implications for developing sex‐specific therapeutic strategies.

Beyond revealing distinct phenotypic patterns, the significantly prolonged hospitalization in sarcopenic patients (9.00 vs. 7.00 days, *p* < 0.001) was observed, aligns with extensive literature linking sarcopenia to adverse postoperative outcomes [7, 8]. The multicompartment depletion, reduced BMR and cellular dysfunction revealed by BIA provide a plausible physiological basis for this delayed recovery. Reassuringly, such alterations may be modifiable. Accumulating evidence from both preclinical and clinical studies indicates that targeted interventions, including dietary modification, nutritional supplementation and structured exercise, can effectively counteract muscle atrophy and improve physical function [38, 39]. The AWGS 2019 consensus recommends a daily protein intake of ≥ 1.2 g/kg and supplementation with essential amino acids or β‐hydroxy‐β‐methylbutyrate for sarcopenic individuals [12]. Building on this and informed by our discovery of sex‐specific phenotypes, we propose a move beyond generic prehabilitation toward sex‐tailored strategies. For males, whose sarcopenia primarily presents as isolated muscle loss with preserved adiposity, an aggressive anabolic protocol is suggested. This could combine high‐intensity resistance training with high‐dose protein or essential amino acid supplementation to directly stimulate muscle protein synthesis. For females, who exhibit coordinated multicompartment atrophy (muscle, fat and bone) and pronounced cellular dysfunction, a more integrated regimen is warranted. This should merge moderate resistance and aerobic exercise with comprehensive, energy‐dense nutritional support. Furthermore, the cellular phenotype suggests exploring adjuncts that support mitochondrial function and membrane integrity, such as omega‐3 fatty acids and antioxidants [39].

Several limitations of this study should be acknowledged. First, its case‐control design precludes causal inference, and residual confounding from unmeasured variables (e.g., detailed diet or genetics) persists. Second, the sample size limited subgroup statistical power, necessitating the combination of sarcopenia severity groups. Although the inclusion of Stage I–II disease introduces tumour burden heterogeneity, this was adjusted for in our models. Third, the use of Asian‐specific diagnostic criteria enhances internal validity for the target population but may affect generalizability to other ethnic groups. Additionally, the high proportion of never‐smokers—characteristic of certain Asian and female lung cancer cohorts—may limit extrapolation to populations with a higher smoking prevalence. Finally, while BIA is practical and functionally informative, its accuracy for absolute tissue quantification is inferior to gold‐standard methods like CT or DXA.

Nonetheless, key methodological strengths underpin the findings. First, unlike traditional retrospective studies, our analysis was grounded in a prospectively maintained database with standardized protocols, which included comprehensive BC assessment, objective functional tests and patient‐reported outcomes; this rigorous approach significantly bolsters the reliability and real‐world relevance of our data. Second, the application of a robust PSM strategy that controlled for nine key clinical confounders greatly strengthened the internal validity of our comparisons.

## 5 Conclusion

This study, leveraging standardized, prospectively collected data and rigorous PSM analysis, established that preoperative sarcopenia was a multifaceted syndrome in early‐stage NSCLC, with a relatively high prevalence (12.6%) and a significant association with prolonged hospitalization. BC profiling further uncovered a striking sex‐specific phenotypic difference: Males present with isolated myopenia, whereas females exhibit a parallel decline across muscle, fat and mineral compartments. These findings underscore that sarcopenia is not a single entity but a sex‐specific phenotype. Therefore, integrating BIA into clinical workflows enables nuanced risk assessment and the subsequent design of targeted, sex‐tailored prehabilitation. This is a critical step toward improving postoperative outcomes and advancing the paradigm of personalized surgical oncology.

## Ethics Statement

This study was approved by the Research Ethics Committee of the First Affiliated Hospital of Anhui Medical University (approval number: 82240150) and The First Affiliated Hospital of University of Science and Technology of China (approval number: 2025KY‐200). This study was conducted in accordance with the Declaration of Helsinki and the Chinese Ethical Guidelines for Medical and Health Research Involving Human Subjects. All participants provided written informed consent prior to study enrollment.

## Conflicts of Interest

The authors declare no conflicts of interest.

## Supporting information


**Table S1:** Standardized mean differences for covariates before and after PSM between sarcopenia and nonsarcopenia groups.
**Table S2:** Association between multiple BC parameters and sarcopenia stratified by sex.
**Table S3:** Association between multiple BC parameters and sarcopenia stratified by age.
**Table S4:** Association between multiple BC parameters and sarcopenia stratified by nutritional status.
**Table S5:** Comparison of demographic, clinical and BC parameters in propensity‐matched early‐stage NSCLC patients stratified by nonsarcopenia, sarcopenia and severe sarcopenia.
**Table S6:** Multinomial logistic regression analysis of BC parameters associated with sarcopenia and severe sarcopenia in propensity‐matched early‐stage NSCLC Patients.
