# Supplementary material for: Body Composition in Resectable Non‐Small Cell Lung Cancer Patients With Preoperative Sarcopenia: A Propensity‐Matched Analysis
Source: J Cachexia Sarcopenia Muscle. 2026 Feb 12;17(1):e70230. doi: 10.1002/jcsm.70230 (PMC12902443; doi:10.1002/jcsm.70230)
Supplement: Supplementary file 1 — Table S1: Standardized mean differences for covariates before and after PSM between sarcopenia and nonsarcopenia groups. Table S2: Association between multiple BC parameters and sarcopenia stratified by sex. Table S3: Association between multiple BC parameters and sarcopenia stratified by age. Table S4: Association between multiple BC parameters and sarcopenia stratified by nutritional status. Table S5: Comparison of demographic, clinical and BC parameters in propensity‐matched early‐stage NSCLC patients stratified by nonsarcopenia, sarcopenia and severe sarcopenia. Table S6: Multinomial logistic regression analysis of BC parameters associated with sarcopenia and severe sarcopenia in propensity‐matched early‐stage NSCLC Patients. [file JCSM-17-e70230-s001.docx]

**Supplementary Materials**

**Supplementary Table S1** Standardized mean differences for covariates before and after PSM between sarcopenia and non-sarcopenia groups.

**Supplementary Table S2** Association between multiple BC parameters and sarcopenia stratified by sex.

**Supplementary Table S3** Association between multiple BC parameters and sarcopenia stratified by age.

**Supplementary Table S4** Association between multiple BC parameters and sarcopenia stratified by nutritional status.

**Supplementary Table S5** Comparison of demographic, clinical, and BC parameters in propensity-matched early-stage NSCLC patients stratified by non-sarcopenia, sarcopenia and severe sarcopenia.

**Supplementary Table S6** Multinomial Logistic Regression Analysis of BC Parameters Associated with Sarcopenia and Severe Sarcopenia in Propensity-Matched Early-Stage NSCLC Patients.

| **Supplementary Table S1** Standardized mean differences for covariates before and after PSM between sarcopenia and non-sarcopenia groups. | | |
| --- | --- | --- |
| **Variable** | **SMD Before Matching** | **SMD After Matching** |
| distance | 0.86 | 0.01 |
| Age Group (<50 years) | -0.11 | 0.02 |
| Age Group (50-59years) | -0.23 | -0.03 |
| Age Group (60-69years) | 0.10 | -0.02 |
| Age Group (70^+^years) | 0.24 | ˂0.01 |
| Sex (Male) | 0.07 | 0.01 |
| Height | -0.38 | -0.04 |
| Extent of resection (Lobectomy) | 0.22 | ˂0.01 |
| Extent of resection (Segmentectomy) | -0.18 | ˂0.01 |
| Extent of resection (Sublobar) | -0.12 | ˂0.01 |
| Extent of resection (Wedge resection) | 0.07 | ˂0.01 |
| Histology (Squamous Cell Carcinoma) | 0.01 | -0.02 |
| Clinical Stage (II) | 0.08 | ˂0.01 |
| PA Levels (Low) | -0.03 | -0.04 |
| PA Levels (Moderate) | -0.03 | 0.07 |
| PA_Levels (High) | 0.07 | -0.03 |
| Smoking history (Current Smoker) | 0.12 | -0.01 |
| Smoking history (Former Smoker) | 0.03 | -0.02 |
| Smoking history (Non-smoker) | -0.14 | 0.01 |
| mPG-SGA category (Stage B or C) | 0.11 | 0.01 |
| Diabetes | -0.05 | 0.02 |
| NAFLD | -0.08 | 0.02 |
| Abbreviation: SMD, standardized mean differences; PA, physical activity; mPG-SGA, modified patient-generated subjective global assessment; NAFLD, nonalcoholic fatty liver disease. | | |

| **Supplementary Table S2** Association between multiple BC parameters and sarcopenia stratified by sex. | | | |
| --- | --- | --- | --- |
| **Parameters** | **Female (n=115)** | **Male (n=94)** | ***P* for interaction** |
| BMI | 0.37 (0.22, 0.62) | 0.49 (0.36, 0.65) | 0.353 |
| CC | 0.58 (0.45, 0.76) | 0.70 (0.55, 0.90) | 0.342 |
| WHR | 1.66 (0.77, 3.56) | 8.80 (2.36, 32.83) | **0.004** |
| ASM | 0.95 (0.93, 0.97) | 0.97 (0.96, 0.99) | 0.127 |
| FFM | 0.82 (0.75, 0.89) | 0.97 (0.91, 1.02) | **0.001** |
| SLM | 0.71 (0.62, 0.83) | 0.89 (0.82, 0.96) | **0.007** |
| FFMI | 0.20 (0.00, 955.45) | 1.68 (1.06, 2.67) | 0.564 |
| BFM | 0.21 (0.07, 0.59) | 0.50 (0.23, 1.09) | 0.172 |
| VFA | 0.27 (0.14, 0.51) | 0.25 (0.16, 0.40) | 0.804 |
| PBF | 0.47 (0.34, 0.63) | 0.90 (0.74, 1.11) | **<0.001** |
| FMI | 0.30 (0.17, 0.55) | 0.26 (0.17, 0.39) | 0.523 |
| PhA | 0.12 (0.03, 0.48) | 0.36 (0.09, 1.39) | 0.367 |
| BCM | 0.43 (0.00, 4241.73) | 1.87 (1.14, 3.08) | 0.568 |
| BMC | 0.06 (0.01, 0.58) | 0.51 (0.12, 2.24) | 0.170 |
| Protein | 0.19 (0.08, 0.46) | 0.21 (0.09, 0.50) | 0.796 |
| Minerals | 0.96 (0.93, 0.98) | 0.94 (0.91, 0.96) | 0.994 |
| TBW | 0.05 (0.01, 0.36) | 0.00 (0.00, 0.00) | 0.512 |
| ICW | 0.38 (0.23, 0.62) | 0.32 (0.21, 0.49) | 0.462 |
| ECW | 0.36 (0.22, 0.61) | 0.30 (0.19, 0.47) | 0.416 |
| BMR | 0.72 (0.27, 1.89) | 1.20 (0.77, 1.87) | 0.300 |
| Adjusted on age group, sex, height, physical activity level, diabetes, nonalcoholic fatty liver disease, histology, clinical stage, modified patient-generated subjective global assessment. Abbreviations: BMI, body mass index; CC, calf circumference; WHR: waist-hip ratio; ASM, appendicular skeletal muscle mass; FFM, fat free mass; SLM, soft lean mass; FFMI, fat free mass index; BFM, body fat mass; VFA, visceral fat area; PBF, percent body fat; FMI, fat mass index; PhA: phase angle; BCM, body cell mass; BMC, bone mineral content; TBW, total body water; ICW, intracellular water; ECW, extracellular water; BMR: Basal metabolic rate. | | | |

| **Supplementary Table S3** Association between multiple BC parameters and sarcopenia stratified by age. | | | |
| --- | --- | --- | --- |
| **Parameters** | **<60 (n=82)** | **≥60 (n=127)** | ***P* for interaction** |
| BMI | 0.34 (0.19, 0.63) | 0.46 (0.34, 0.62) | 0.352 |
| Calf circumference | 0.60 (0.42, 0.87) | 0.63 (0.50, 0.79) | 0.940 |
| WHR | 2.65 (1.06, 6.62) | 3.28 (1.56, 6.90) | 0.909 |
| ASM | 0.95 (0.92, 0.99) | 0.96 (0.95, 0.98) | 0.613 |
| FFM | 0.87 (0.79, 0.97) | 0.90 (0.85, 0.95) | 0.574 |
| SLM | 0.76 (0.62, 0.94) | 0.83 (0.77, 0.89) | 0.431 |
| FFMI | 1.59 (0.88, 2.86) | 1.49 (0.90, 2.44) | 0.879 |
| BFM | 0.34 (0.13, 0.89) | 0.32 (0.16, 0.66) | 0.888 |
| VFA | 0.22 (0.10, 0.48) | 0.24 (0.13, 0.46) | 0.695 |
| PBF | 0.64 (0.42, 0.97) | 0.69 (0.58, 0.83) | 0.709 |
| FMI | 0.25 (0.13, 0.49) | 0.27 (0.13, 0.53) | 0.880 |
| PhA | 0.26 (0.04, 1.61) | 0.18 (0.06, 0.50) | 0.997 |
| BCM | 1.73 (0.97, 3.08) | 1.63 (1.04, 2.57) | 0.834 |
| BMC | 0.24 (0.04, 1.47) | 0.21 (0.05, 0.87) | 0.796 |
| Protein | 0.18 (0.06, 0.53) | 0.16 (0.07, 0.37) | 0.656 |
| Minerals | 0.95 (0.92, 0.97) | 0.96 (0.93, 0.98) | 0.833 |
| TBW | 0.03 (0.00, 0.24) | 0.03 (0.00, 0.21) | 0.643 |
| ICW | 0.31 (0.16, 0.60) | 0.37 (0.21, 0.64) | 0.988 |
| ECW | 0.29 (0.14, 0.60) | 0.35 (0.20, 0.62) | 0.938 |
| BMR | 1.41 (0.61, 3.24) | 0.99 (0.55, 1.79) | 0.895 |
| Adjusted on age group, sex, height, physical activity level, diabetes, nonalcoholic fatty liver disease, histology, clinical stage, modified patient-generated subjective global assessment. Abbreviations: BMI, body mass index; CC, calf circumference; WHR: waist-hip ratio; ASM, appendicular skeletal muscle mass; FFM, fat free mass; SLM, soft lean mass; FFMI, fat free mass index; BFM, body fat mass; VFA, visceral fat area; PBF, percent body fat; FMI, fat mass index; PhA: phase angle; BCM, body cell mass; BMC, bone mineral content; TBW, total body water; ICW, intracellular water; ECW, extracellular water; BMR: Basal metabolic rate. | | | |

| **Supplementary Table S4** Association between multiple BC parameters and sarcopenia stratified by nutritional status. | | | |
| --- | --- | --- | --- |
| **Parameters** | **Well-nourished or Mild Malnutrition (n=177)** | **Moderate or Severe Malnutrition (n=32)** | ***P*** for interaction |
| BMI | 0.41 (0.30, 0.57) | 0.38 (0.20, 0.75) | 0.899 |
| Calf circumference | 0.62 (0.49, 0.77) | 0.61 (0.39, 0.94) | 0.680 |
| WHR | 3.44 (1.86, 6.40) | 0.00 (0.00, 13.82) | 0.098 |
| ASM | 0.96 (0.94, 0.97) | 0.97 (0.95, 1.00) | 0.154 |
| FFM | 0.89 (0.84, 0.94) | 0.90 (0.77, 1.04) | 0.306 |
| SLM | 0.80 (0.73, 0.87) | 0.80 (0.66, 0.97) | 0.423 |
| FFMI | 1.45 (1.01, 2.08) | 0.00 (0.00, 0.32) | 0.075 |
| BFM | 0.39 (0.19, 0.78) | 0.04 (0.01, 0.25) | 0.106 |
| VFA | 0.29 (0.17, 0.50) | 0.13 (0.04, 0.41) | 0.880 |
| PBF | 0.68 (0.55, 0.83) | 0.54 (0.33, 0.91) | 0.869 |
| FMI | 0.30 (0.18, 0.51) | 0.24 (0.11, 0.51) | 0.757 |
| PhA | 0.25 (0.08, 0.84) | 0.05 (0.01, 0.37) | 0.342 |
| BCM | 1.60 (1.11, 2.30) | 0.00 (0.00, 0.30) | 0.089 |
| BMC | 0.30 (0.07, 1.33) | 0.00 (0.00, 0.08) | 0.255 |
| Protein | 0.22 (0.10, 0.50) | 0.05 (0.01, 0.31) | 0.719 |
| Minerals | 0.95 (0.93, 0.98) | 0.95 (0.92, 0.97) | 0.846 |
| TBW | 0.03 (0.00, 0.24) | 0.02 (0.00, 0.20) | 0.129 |
| ICW | 0.37 (0.24, 0.58) | 0.36 (0.21, 0.61) | 0.848 |
| ECW | 0.35 (0.22, 0.56) | 0.34 (0.19, 0.59) | 0.836 |
| BMR | 1.10 (0.72, 1.66) | 0.31 (0.05, 1.99) | 0.305 |
| Adjusted on age group, sex, height, physical activity level, diabetes, nonalcoholic fatty liver disease, histology, clinical stage, modified patient-generated subjective global assessment. Abbreviations: BMI, body mass index; CC, calf circumference; WHR: waist-hip ratio; ASM, appendicular skeletal muscle mass; FFM, fat free mass; SLM, soft lean mass; FFMI, fat free mass index; BFM, body fat mass; VFA, visceral fat area; PBF, percent body fat; FMI, fat mass index; PhA: phase angle; BCM, body cell mass; BMC, bone mineral content; TBW, total body water; ICW, intracellular water; ECW, extracellular water; BMR: Basal metabolic rate. | | | |

| **Supplementary Table S5** Comparison of demographic, clinical, and BC parameters in propensity-matched early-stage NSCLC patients stratified by non-sarcopenia, sarcopenia and severe sarcopenia. | | | | |
| --- | --- | --- | --- | --- |
| **Variables** | **non-Sarcopenia (n=162)** | **Sarcopenia (n=27)** | **Severe Sarcopenia (n=20)** | ***P* value** |
| **Demographic and Clinical Characteristics** | | | | |
| Age Group (n, %)) |  |  |  | 0.020 ^b^ |
| <50 years | 32 (19.8) | 6 (22.2) | 1 (5.0) |  |
| 50-59years | 35 (21.6) | 7 (25.9) | 1 (5.0) |  |
| 60-69years | 59 (36.4) | 10 (37.0) | 6 (30.0) |  |
| 70+years | 36 (22.2) | 4 (14.8) | 12 (60.0) |  |
| Sex (%) |  |  |  | 0.889 ^a^ |
| Female | 88 (54.3) | 15 (55.6) | 12 (60.0) |  |
| Male | 74 (45.7) | 12 (44.4) | 8 (40.0) |  |
| Education Level (n, %) |  |  |  | 0.065 ^b^ |
| Illiterate or Primary School | 79 (48.8) | 10 (37.0) | 14 (70.0) |  |
| Junior High School | 39 (24.1) | 8 (29.6) | 4 (20.0) |  |
| Senior High School | 17 (10.5) | 7 (25.9) | 2 (10.0) |  |
| College or higher |  |  |  |  |
| Marital Status (n, %)) |  |  |  | 0.005 ^b^ |
| Married | 158 (97.5) | 25 (92.6) | 16 (80.0) |  |
| Single, divorced, or widowed | 4 (2.5) | 2 (7.4) | 4 (20.0) |  |
| Residence (n, %) |  |  |  | 0.353 ^b^ |
| Municipalities | 58 (35.8) | 8 (29.6) | 4 (20.0) |  |
| Rural | 78 (48.1) | 12 (44.4) | 10 (50.0) |  |
| Townships |  |  |  |  |
| Smoking History (n, %) |  |  |  | 0.012 ^b^ |
| Current Smoker | 24 (14.8) | 0 (0.0) | 6 (30.0) |  |
| Former Smoker | 26 (16.0) | 8 (29.6) | 1 (5.0) |  |
| Non-Smoker | 112 (69.1) | 19 (70.4) | 13 (65.0) |  |
| Alcohol Consumption Status (n, %) |  |  |  | 0.178 ^b^ |
| Current Drinker | 40 (24.7) | 3 (11.1) | 5 (25.0) |  |
| Former Drinker | 22 (13.6) | 5 (18.5) | 0 (0.0) |  |
| Non-Drinker | 100 (61.7) | 19 (70.4) | 15 (75.0) |  |
| Green Tea Drinking (n, %) |  |  |  | 0.143 ^a^ |
| No | 77 (47.5) | 15 (55.6) | 14 (70.0) |  |
| Yes | 85 (52.5) | 12 (44.4) | 6 (30.0) |  |
| Diabetes (n, %) | 14 (8.6) | 1 (3.7) | 2 (10.0) | 0.667 ^b^ |
| NAFLD (n, %) | 16 (9.9) | 1 (3.7) | 1 (5.0) | 0.615 ^b^ |
| Sedentary Time, min | 240.00 [120.00, 300.00] | 240.00 [180.00, 300.00] | 240.00 [60.00, 270.00] | 0.633 ^c^ |
| Physical Activity Level (n, %) |  |  |  | 0.772 ^b^ |
| Low | 20 (12.3) | 3 (11.1) | 3 (15.0) |  |
| Moderate | 59 (36.4) | 8 (29.6) | 9 (45.0) |  |
| High | 83 (51.2) | 16 (59.3) | 8 (40.0) |  |
| Histology (n, %) |  |  |  | 0.639 ^b^ |
| Adenocarcinoma | 157 (96.9) | 26 (96.3) | 19 (95.0) |  |
| Squamous Cell Carcinoma | 5 (3.1) | 1 (3.7) | 1 (5.0) |  |
| Clinical Stage (n, %) |  |  |  | 0.400 ^b^ |
| I | 161 (99.4) | 26 (96.3) | 20 (100.0) |  |
| II | 1 (0.6) | 1 (3.7) | 0 (0.0) |  |
| Side of Pneumonectomy (n, %) |  |  |  | 0.754 ^a^ |
| Left | 60 (37.0) | 12 (44.4) | 8 (40.0) |  |
| Right | 102 (63.0) | 15 (55.6) | 12 (60.0) |  |
| Extent of Resection (n, %) |  |  |  | 0.881 ^b^ |
| Lobectomy | 77 (47.5) | 11 (40.7) | 11 (55.0) |  |
| Segmentectomy | 11 (6.8) | 1 (3.7) | 2 (10.0) |  |
| Sublobar | 34 (21.0) | 6 (22.2) | 3 (15.0) |  |
| Wedge resection | 40 (24.7) | 9 (33.3) | 4 (20.0) |  |
| **Laboratory Parameters** |  |  |  |  |
| Hemoglobin, g/L | 138.00 [128.00, 148.00] | 137.00 [129.00, 147.00] | 126.50 [114.50, 137.00] | 0.014 ^c^ |
| Prealbumin, g/L | 271.50 [229.25, 318.00] | 247.00 [226.00, 281.00] | 201.00 [170.00, 239.50] | <0.001 ^c^ |
| Albumin, g/L | 44.90 [42.62, 46.80] | 44.50 [43.55, 47.65] | 42.20 [39.15, 43.75] | 0.002 ^c^ |
| Globulin, g/L | 26.50 [24.20, 29.20] | 26.90 [24.90, 28.25] | 25.60 [22.12, 27.42] | 0.154 ^c^ |
| Absolute Neutrophil Count, ×10^9^/L | 3.49 [2.69, 4.38] | 3.18 [2.49, 3.60] | 3.51 [2.42, 5.29] | 0.166 ^c^ |
| Lymphocyte Count, ×10^9^/L | 1.79 [1.49, 2.25] | 1.61 [1.39, 2.04] | 1.34 [1.14, 2.04] | 0.059 ^c^ |
| **Surgical Outcomes** |  |  |  |  |
| Length of Hospital Stay, day | 7.00 [5.00, 9.00] | 8.00 [6.00, 12.00] | 10.00 [8.00, 15.25] | 0.003 ^c^ |
| Chest Tube Drainage Time, day | 5.00 [4.00, 6.00] | 5.00 [4.00, 6.50] | 5.00 [4.00, 6.25] | 0.886 ^c^ |
| Drainage Volume in Total, mL | 465.00 [212.50, 938.75] | 400.00 [165.00, 737.50] | 575.00 [271.25, 980.00] | 0.600 ^c^ |
| **Anthropometric Parameters** | | | | |
| Height, m | 1.62 [1.56, 1.67] | 1.63 [1.57, 1.67] | 1.61 [1.53, 1.64] | 0.346 ^c^ |
| Weight, kg | 64.05 [57.05, 72.25] | 49.50 [46.20, 56.35] | 50.65 [43.95, 54.35] | <0.001 ^c^ |
| BMI Category (n, %) |  |  |  | <0.001 ^b^ |
| ˂18.5 | 2 (1.2) | 7 (25.9) | 6 (30.0) |  |
| 18.5-24.9 | 87 (53.7) | 20 (74.1) | 13 (65.0) |  |
| >24.9 | 73 (45.1) | 0 (0.0) | 1 (5.0) |  |
| mPG-SGA Category (n, %) |  |  |  | 0.416 ^b^ |
| Well-nourished or Mild Malnutrition | 139 (85.8) | 23 (85.2) | 15 (75.0) |  |
| Moderate or Severe Malnutrition | 23 (14.2) | 4 (14.8) | 5 (25.0) |  |
| Calf Circumference, cm | 34.70 ± 3.01 | 31.28 ± 3.21 | 30.11 ± 3.87 | <0.001 ^d^ |
| Handgrip Strength/m^2^ | 10.22 [8.58, 12.44] | 10.47 [8.73, 11.75] | 7.18 [6.65, 8.40] | <0.001 ^c^ |
| Chair Stand Test, sec | 12.40 [10.03, 15.30] | 14.00 [12.57, 15.11] | 18.19 [15.26, 20.72] | <0.001 ^c^ |
| **Body Composition Parameters** | | | | |
| Waist Hip Ratio | 0.91 [0.87, 0.95] | 0.88 [0.84, 0.94] | 0.88 [0.84, 0.91] | 0.116 ^c^ |
| ASM, kg | 18.20 [15.12, 21.67] | 15.00 [13.10, 19.27] | 14.28 [12.14, 17.00] | <0.001 ^c^ |
| Skeletal Muscle Index, kg/m^2^ | 7.00 [6.20, 7.68] | 5.60 [5.45, 6.90] | 5.50 [5.20, 6.12] | <0.001 ^c^ |
| Body Fat Mass, kg | 18.93 ± 6.34 | 13.00 ± 4.14 | 12.45 ± 4.15 | <0.001 ^d^ |
| Fat Free Mass, kg | 44.95 [39.10, 51.70] | 37.50 [34.15, 44.80] | 36.90 [33.85, 42.12] | <0.001 ^c^ |
| FFM of Trunk, kg | 20.70 [18.00, 23.50] | 16.70 [15.15, 21.40] | 16.70 [15.40, 17.75] | <0.001 ^c^ |
| FFM of Right Arm, kg | 2.45 [2.00, 2.96] | 1.76 [1.55, 2.58] | 1.78 [1.59, 1.98] | <0.001 ^c^ |
| FFM of Left Arm, kg | 2.44 [2.01, 2.83] | 1.71 [1.48, 2.49] | 1.80 [1.53, 2.00] | <0.001 ^c^ |
| FFM of Right Leg, kg | 6.75 [5.67, 7.94] | 5.75 [5.04, 7.05] | 5.45 [4.47, 6.21] | <0.001 ^c^ |
| FFM of Left Leg, kg | 6.69 [5.60, 7.91] | 5.76 [4.94, 7.03] | 5.30 [4.53, 6.41] | <0.001 ^c^ |
| Soft Lean Mass, kg | 42.45 [36.80, 49.00] | 35.40 [32.15, 42.35] | 34.75 [31.93, 39.88] | <0.001 ^c^ |
| Visceral Fat Area, cm^2^ | 86.65 [67.00, 115.35] | 52.60 [47.45, 83.00] | 59.20 [41.35, 71.70] | <0.001 ^c^ |
| Percent Body Fat, % | 29.80 [24.02, 34.40] | 23.50 [21.65, 28.15] | 24.70 [20.60, 27.25] | <0.001 ^c^ |
| Fat Free Mass Index, kg/m^2^ | 17.15 [16.10, 18.48] | 14.80 [14.10, 16.70] | 14.95 [14.12, 16.20] | <0.001 ^c^ |
| Fat Mass Index, kg/m^2^ | 7.29 ± 2.61 | 5.42 ± 1.78 | 5.34 ± 2.34 | <0.001 ^d^ |
| Phase Angle, ° | 5.30 [4.80, 5.90] | 5.20 [4.75, 5.70] | 4.75 [4.30, 5.10] | 0.015 ^c^ |
| Body Cell Mass, kg | 29.10 [25.30, 33.70] | 24.30 [22.20, 29.15] | 23.60 [22.10, 28.10] | <0.001 ^c^ |
| Bone Mineral Content, kg | 2.52 [2.22, 2.79] | 2.36 [2.04, 2.64] | 2.21 [1.94, 2.47] | 0.008 |
| Protein, kg | 8.80 [7.60, 10.20] | 7.50 [6.70, 9.10] | 7.15 [6.72, 8.75] | <0.001 ^c^ |
| Minerals, kg | 3.05 [2.69, 3.43] | 2.86 [2.46, 3.21] | 2.71 [2.38, 3.02] | 0.006 |
| Total Body Water, kg | 33.05 [28.72, 38.38] | 27.70 [25.00, 33.00] | 27.15 [24.80, 31.90] | <0.001 ^c^ |
| Extracellular Water, kg | 12.80 [11.10, 14.78] | 10.70 [9.60, 13.20] | 10.80 [9.47, 13.03] | <0.001 ^c^ |
| Intracellular Water, kg | 20.30 [17.70, 23.50] | 17.30 [15.30, 20.35] | 16.45 [15.38, 20.12] | <0.001 ^c^ |
| ECW/ICW, % | 0.63 [0.62, 0.64] | 0.63 [0.62, 0.65] | 0.65 [0.64, 0.67] | <0.001 ^c^ |
| Basal Metabolic Rate, kcal | 1340.50 [1214.00, 1486.75] | 1134.00 [1107.50, 1337.00] | 1154.00 [1100.50, 1243.25] | <0.001 ^c^ |
| Abbreviations: NAFLD, non-alcoholic fatty liver disease; BMI, body mass index; mPG-SGA, modified patient-generated subjective global assessment; ASM, appendicular skeletal muscle mass; FFM, fat free mass; ECW, extracellular water; ICW, intracellular water. ^a^ Chi-square test, ^b^ Fisher’s exact test, ^c^ Mann-Whiney U test, ^d^ two-sample t-test. | | | | |

| **Supplementary Table S6** Multinomial Logistic Regression Analysis of BC Parameters Associated with Sarcopenia and Severe Sarcopenia in Propensity-Matched Early-Stage NSCLC Patients. | | | | | | | |  |
| --- | --- | --- | --- | --- | --- | --- | --- | --- |
| **Variable** | **Sarcopenia** | |  | **Severe Sarcopenia** | |  |  |  |
|  | OR (95%CI) | *P* value |  | OR (95%CI) | *P* value | | | |
| BMI | 0.44 (0.33, 0.59) | <0.001 |  | 0.39 (0.27, 0.56) | <0.001 | | | |
| CC | 0.67 (0.57, 0.80) | <0.001 |  | 0.56 (0.45, 0.71) | <0.001 | | | |
| WHR | 3.24 (1.04, 10.04) | 0.042 |  | 3.49 (1.10, 11.06) | 0.034 | | | |
| ASM | 0.04 (0.01, 0.13) | <0.001 |  | 0.01 (0.00, 0.06) | <0.001 | | | |
| FFM | 0.35 (0.25, 0.50) | <0.001 |  | 0.38 (0.27, 0.55) | <0.001 | | | |
| SLM | 0.33 (0.23, 0.48) | <0.001 |  | 0.36 (0.25, 0.53) | <0.001 | | | |
| FFMI | 0.33 (0.21, 0.52) | <0.001 |  | 0.33 (0.20, 0.56) | <0.001 | | | |
| BFM | 0.82 (0.74, 0.90) | <0.001 |  | 0.81 (0.72, 0.90) | <0.001 | | | |
| VFA | 0.96 (0.95, 0.98) | <0.001 |  | 0.96 (0.94, 0.98) | <0.001 | | | |
| PBF | 0.90 (0.84, 0.96) | 0.002 |  | 0.89 (0.83, 0.95) | 0.001 | | | |
| FMI | 0.69 (0.55, 0.85) | 0.001 |  | 0.67 (0.53, 0.85) | 0.001 | | | |
| PhA | 1.04 (0.65, 1.66) | 0.872 |  | 1.09 (0.66, 1.80) | 0.737 | | | |
| BCM | 0.25 (0.16, 0.40) | <0.001 |  | 0.28 (0.17, 0.45) | <0.001 | | | |
| BMC | 1.45 (0.88, 2.39) | 0.140 |  | 1.69 (1.05, 2.74) | 0.032 | | | |
| Protein | 0.16 (0.08, 0.36) | <0.001 |  | 0.32 (0.15, 0.69) | 0.004 | | | |
| Mineral | 1.31 (0.80, 2.13) | 0.284 |  | 1.57 (0.99, 2.48) | 0.055 | | | |
| TBW | 0.27 (0.17, 0.42) | <0.001 |  | 0.31 (0.20, 0.48) | <0.001 | | | |
| ICW | 0.18 (0.10, 0.32) | <0.001 |  | 0.21 (0.12, 0.38) | <0.001 | | | |
| ECW | 0.15 (0.07, 0.31) | <0.001 |  | 0.31 (0.16, 0.58) | <0.001 | | | |
| BMR | 0.95 (0.95, 0.95) | <0.001 |  | 0.95 (0.95, 0.96) | <0.001 | | | |
| Reference: Non-Sarcopenia Group. Adjusted on age group, sex, height, physical activity level, diabetes, nonalcoholic fatty liver disease, histology, clinical stage, modified patient-generated subjective global assessment. Abbreviations: BMI, body mass index; CC, calf circumference; WHR: waist-hip ratio; ASM, appendicular skeletal muscle mass; FFM, fat free mass; SLM, soft lean mass; FFMI, fat free mass index; BFM, body fat mass; VFA, visceral fat area; PBF, percent body fat; FMI, fat mass index; PhA, phase angle; BCM, body cell mass; BMC, bone mineral content; TBW, total body water; ICW, intracellular water; ECW, extracellular water; BMR: Basal metabolic rate. | | | | | | |  |  |
